# Supplementary material for: Modern Metaproteomics: A Unique Tool to Characterize the Active Microbiome in Health and Diseases, and Pave the Road towards New Biomarkers—Example of Crohn’s Disease and Ulcerative Colitis Flare-Ups
Source: Cells. 2022 Apr 14;11(8):1340. doi: 10.3390/cells11081340 (PMC9028112; doi:10.3390/cells11081340)
Supplement: Supplementary file 1 [file cells-11-01340-s001.zip › Figure_S1_biblio.pdf]

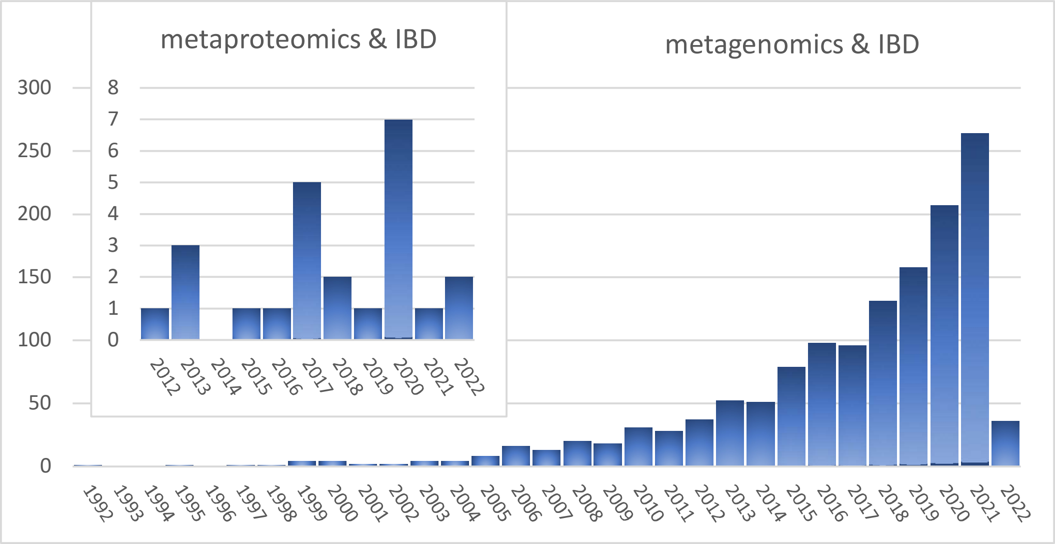

Figure S1. Number of metagenomics and metaproteomics studies related to inflammatory bowel diseases identified in pubmed.ncbi over the 30 last years. Search query for metagenomics: (((IBD[Title/Abstract]) OR (inflammatory bowel disease[Title/Abstract]) OR (Crohn[Title/Abstract]) OR (ulcerative colitis[Title/Abstract])) AND (((metagenomics[Title/Abstract]) OR (shotgun sequencing[Title/Abstract]) OR (16S[Title/Abstract]))). Search query for metaproteomics: Search query: (((IBD[Title/Abstract]) OR (inflammatory bowel disease[Title/Abstract]) OR (Crohn[Title/Abstract]) OR (ulcerative colitis[Title/Abstract])) AND ((metagenomics[Title/Abstract]) OR (shotgun sequencing[Title/Abstract]) OR (16S[Title/Abstract]))).
