# Supplementary material for: Modern Metaproteomics: A Unique Tool to Characterize the Active Microbiome in Health and Diseases, and Pave the Road towards New Biomarkers—Example of Crohn’s Disease and Ulcerative Colitis Flare-Ups
Source: Cells. 2022 Apr 14;11(8):1340. doi: 10.3390/cells11081340 (PMC9028112; doi:10.3390/cells11081340)
Supplement: Supplementary file 1 [file cells-11-01340-s001.zip › Figure_S3_iPath3.pdf]

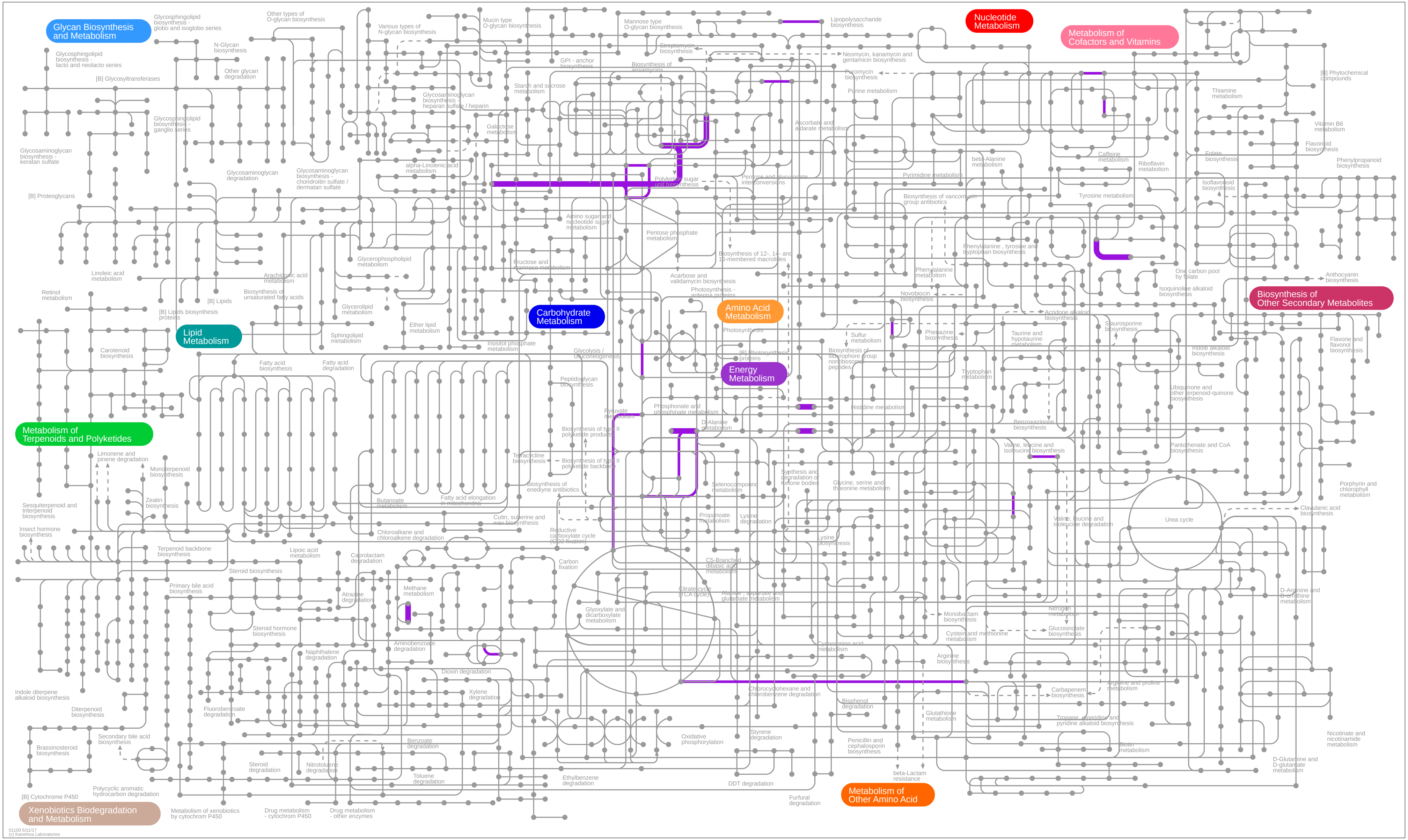

Figure S3a. Metabolic pathways underrepresented in *Proteobacteria* cell envelopes of CDIC microbiota, compared to controls.

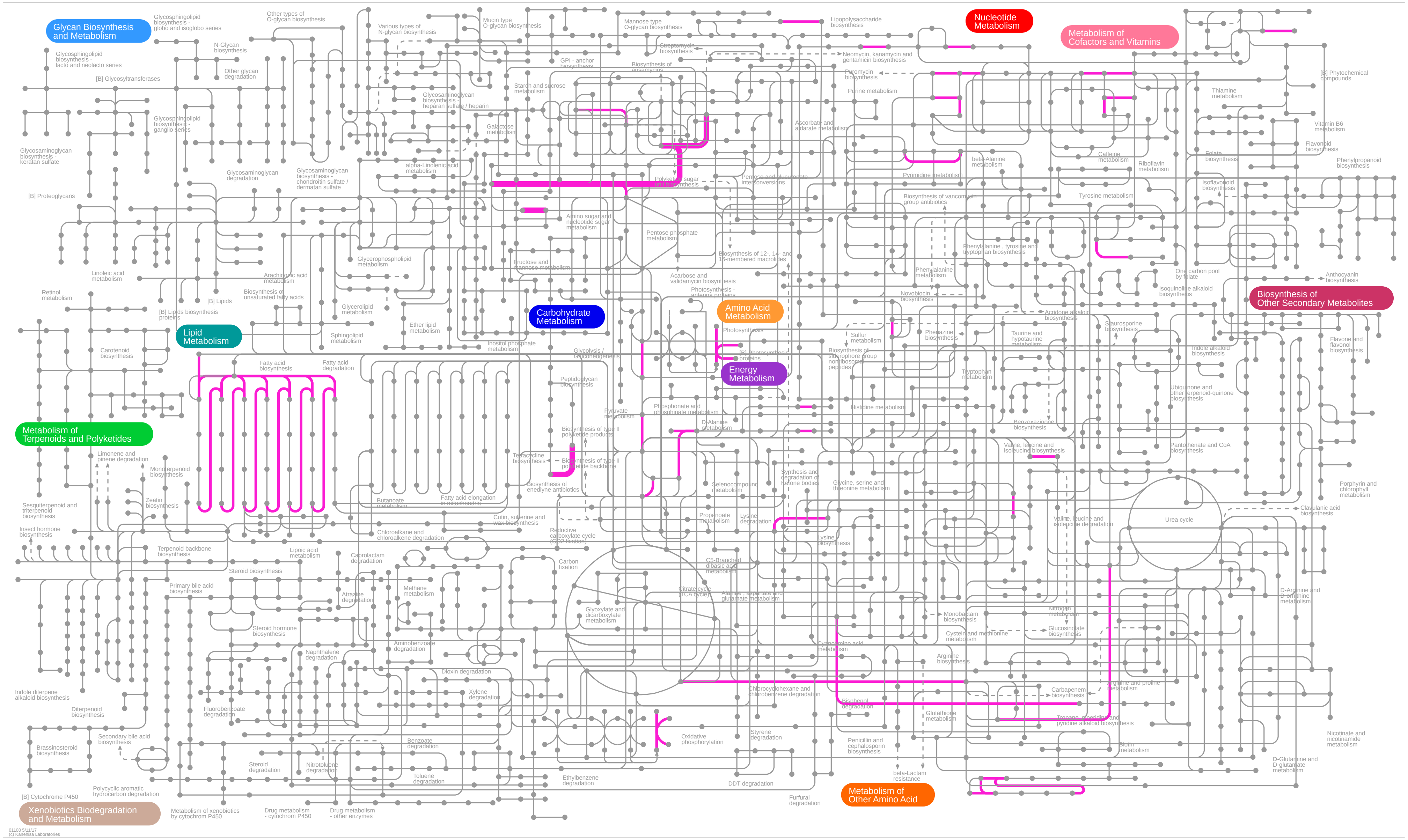

Figure S3b. Metabolic pathways underrepresented in *Proteobacteria* cell envelopes of CDC microbiota, compared to controls.

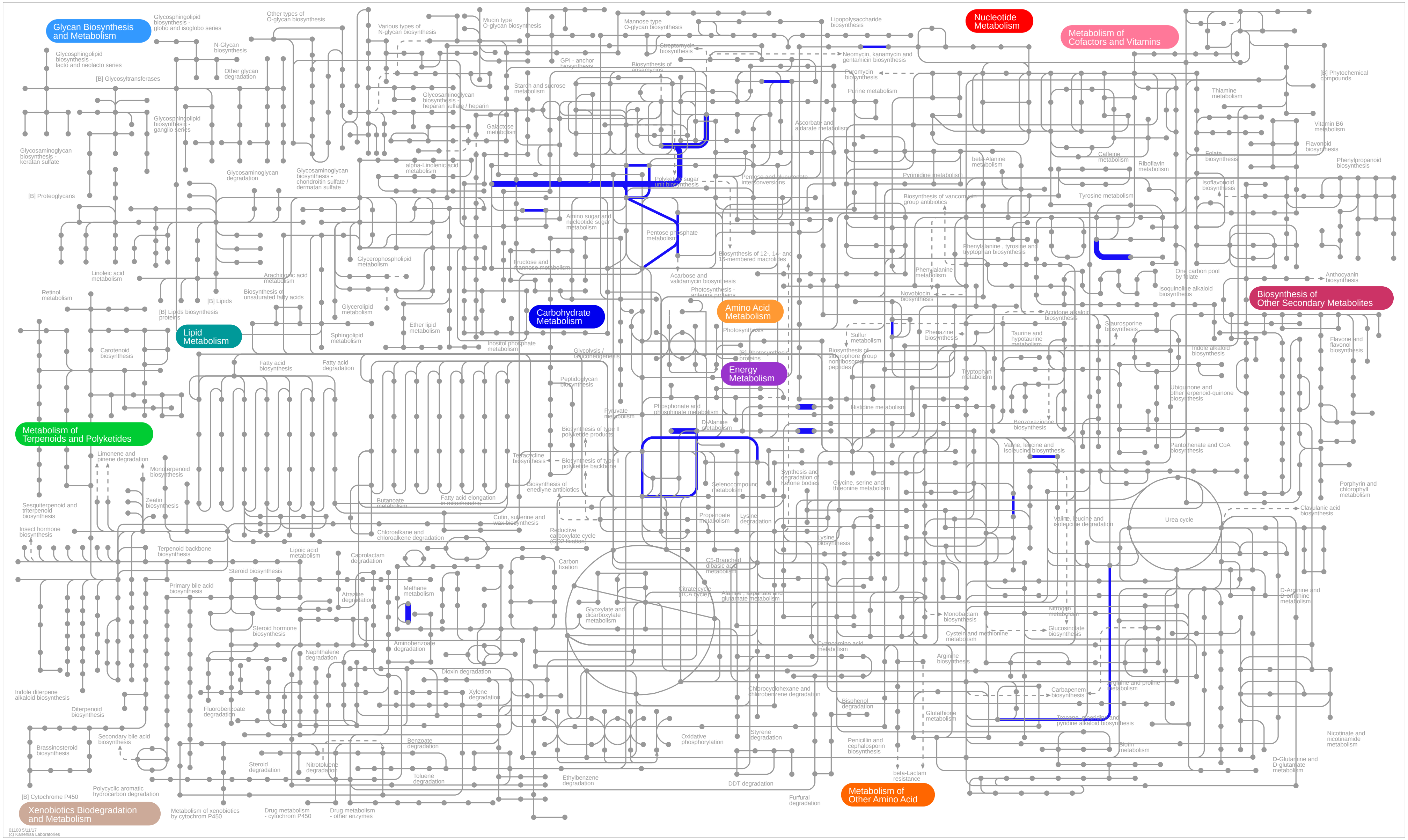

Figure S3c. Metabolic pathways underrepresented in *Proteobacteria* cell envelopes of UC microbiota, compared to controls.

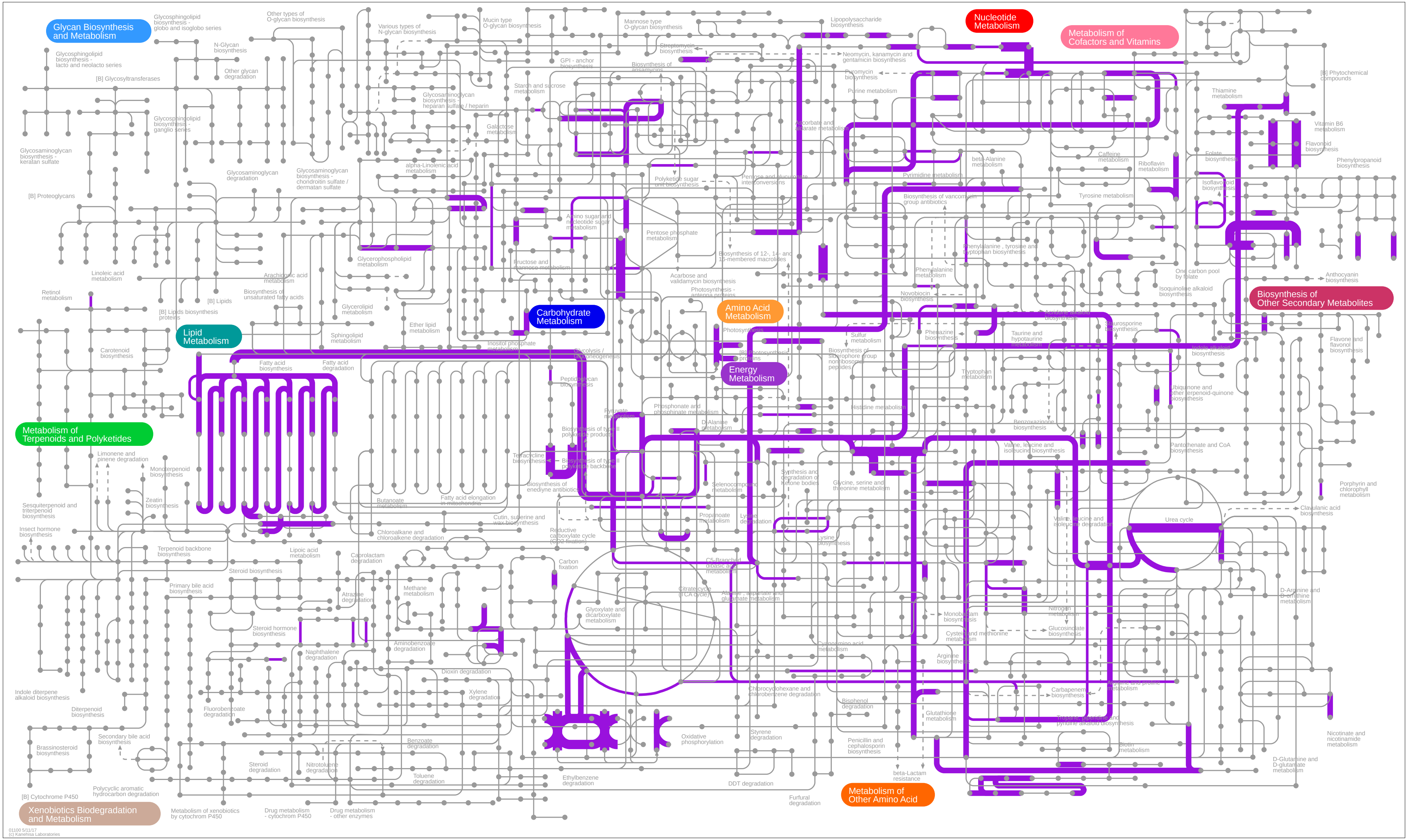

Figure S3d. Metabolic pathways overrepresented in *Proteobacteria* cell envelopes of CDIC microbiota, compared to controls.

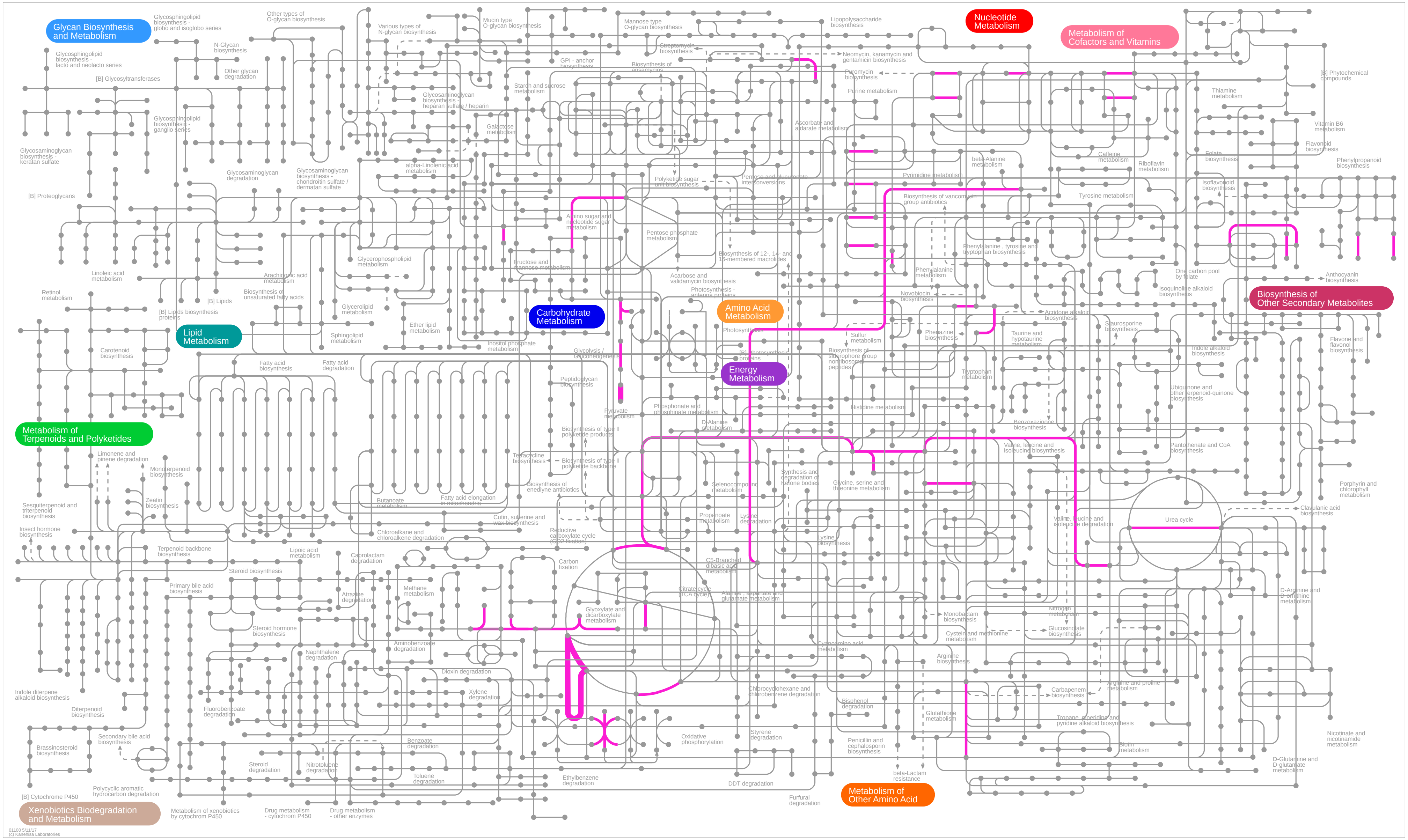

Figure S3e. Metabolic pathways overrepresented in *Proteobacteria* cell envelopes of CDC microbiota, compared to controls.

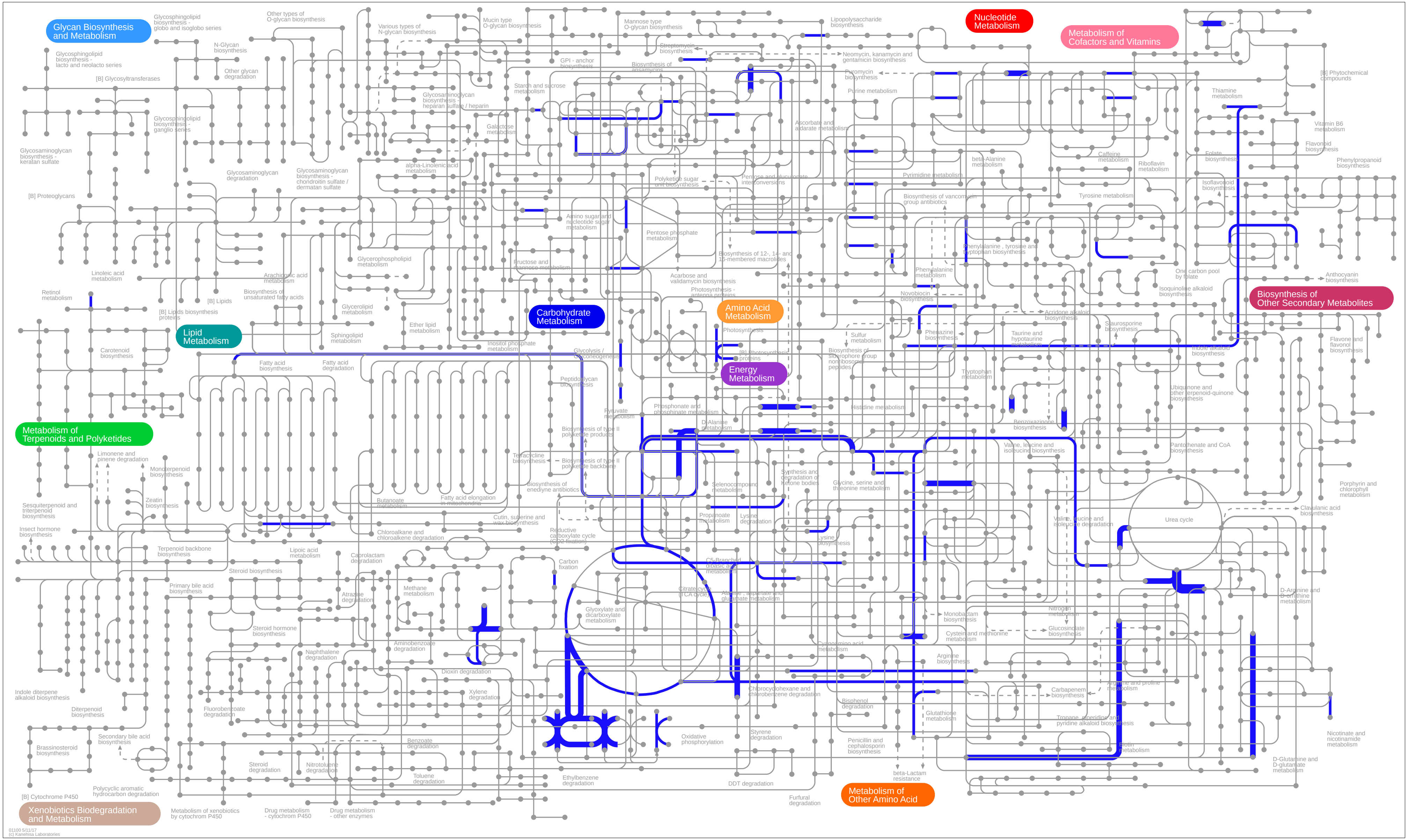

Figure S3f. Metabolic pathways overrepresented in *Proteobacteria* cell envelopes of UC microbiota, compared to controls.

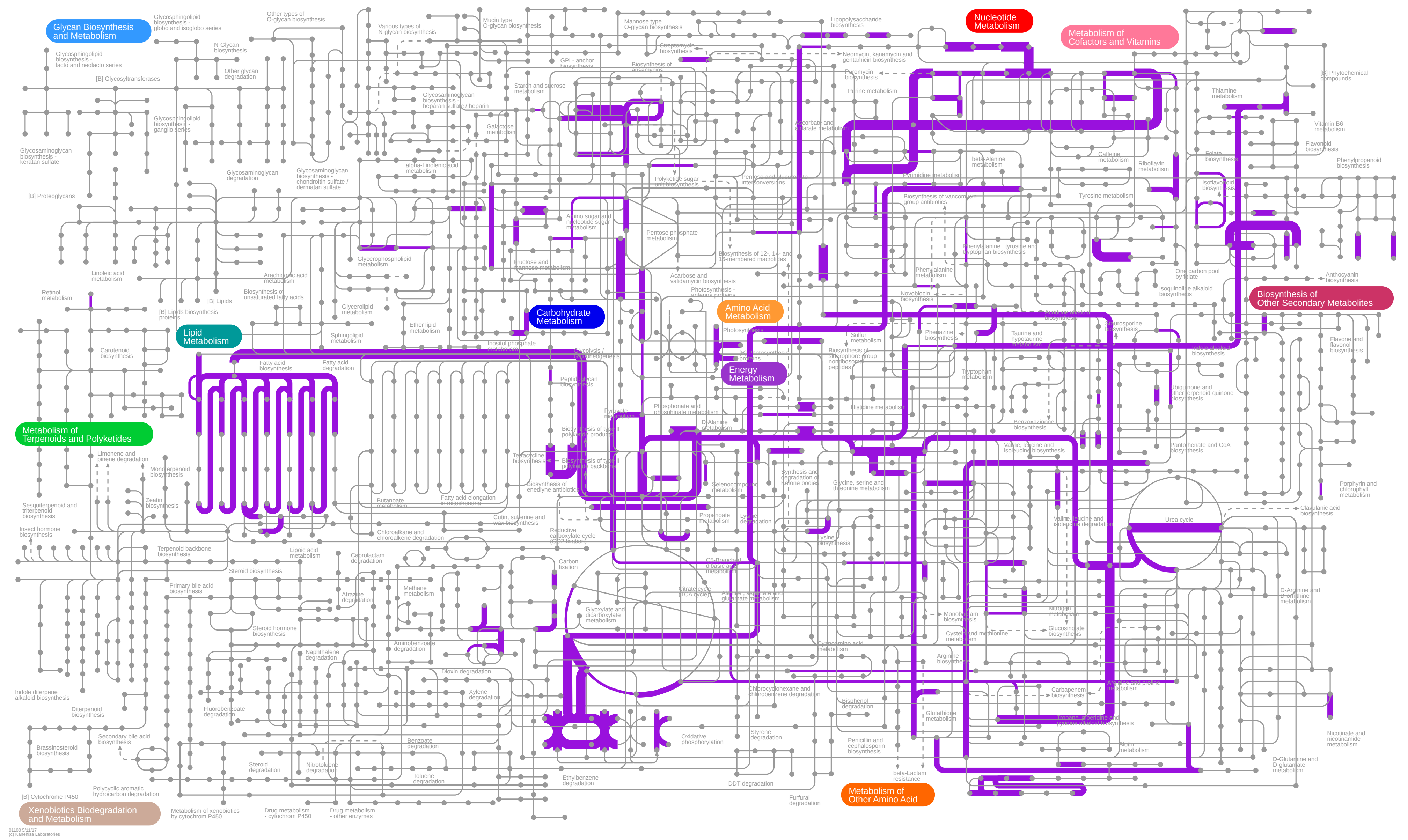

Figure S3g. Metabolic pathways overrepresented in *E. coli* cell envelopes of CDIC microbiota, compared to controls.

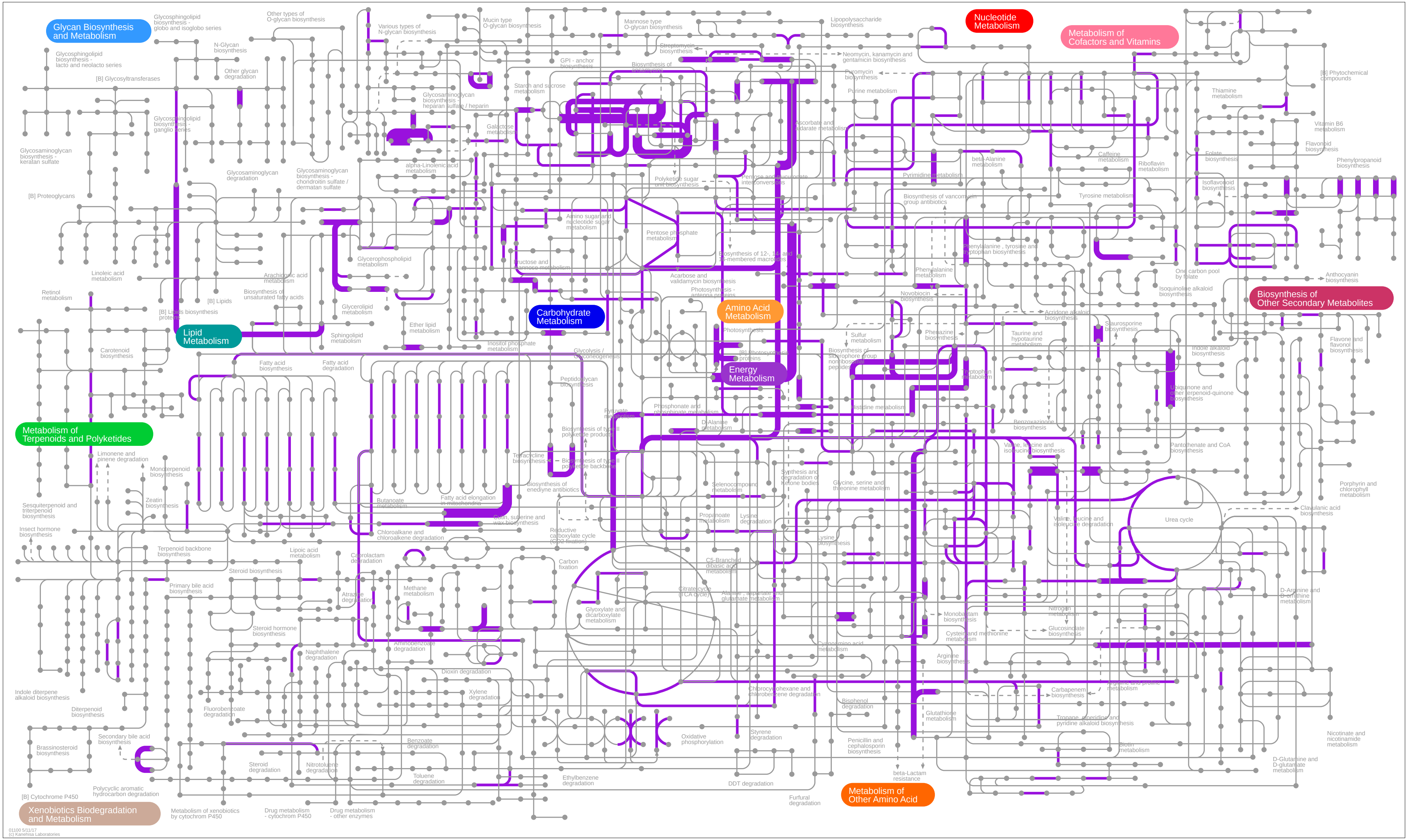

Figure S3h. Metabolic pathways underrepresented in *Firmicutes* cell envelopes of CDIC microbiota, compared to controls.

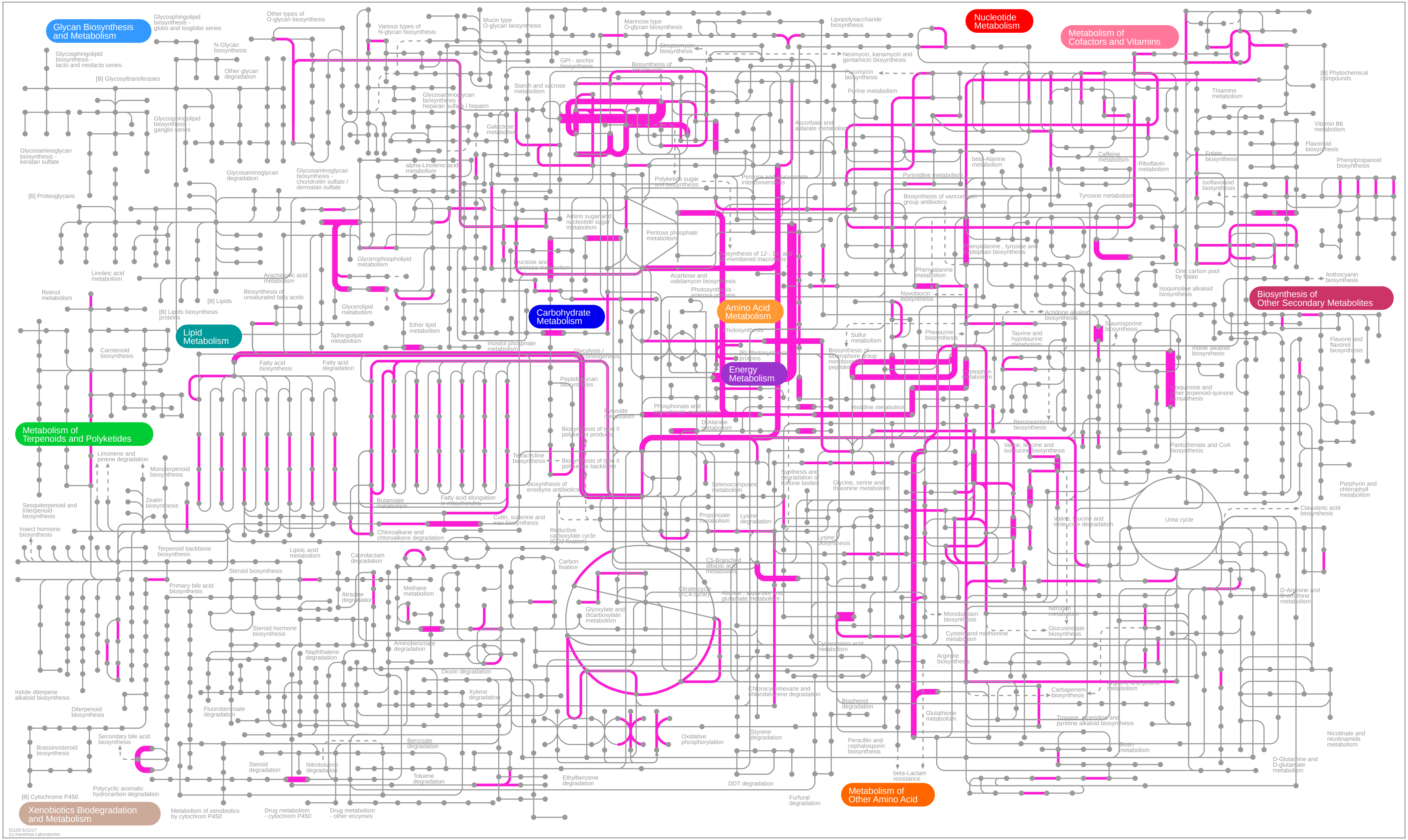

Figure S3i. Metabolic pathways underrepresented in *Firmicutes* cell envelopes of CDC microbiota, compared to controls.

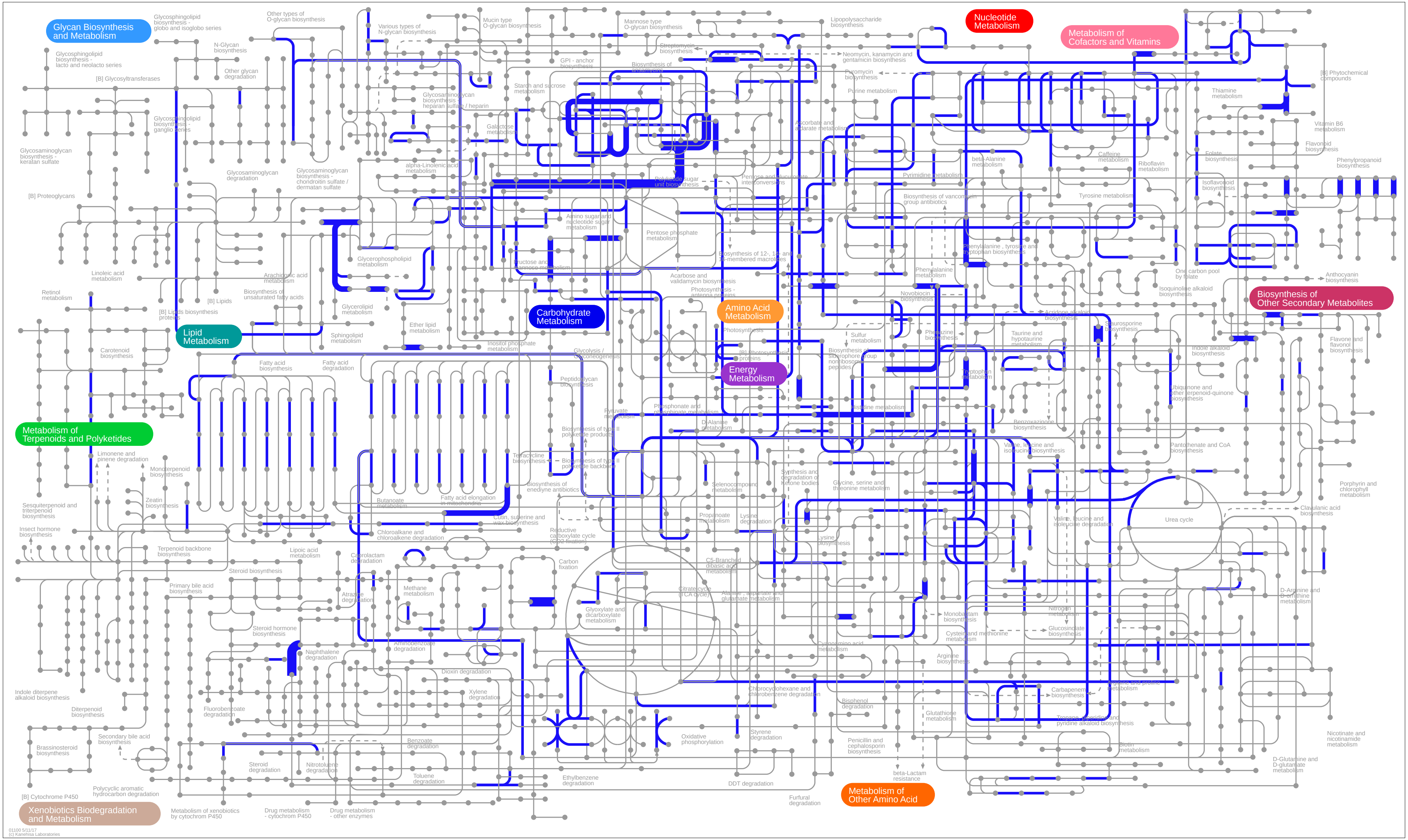

Figure S3j. Metabolic pathways underrepresented in *Firmicutes* cell envelopes of UC microbiota, compared to controls.

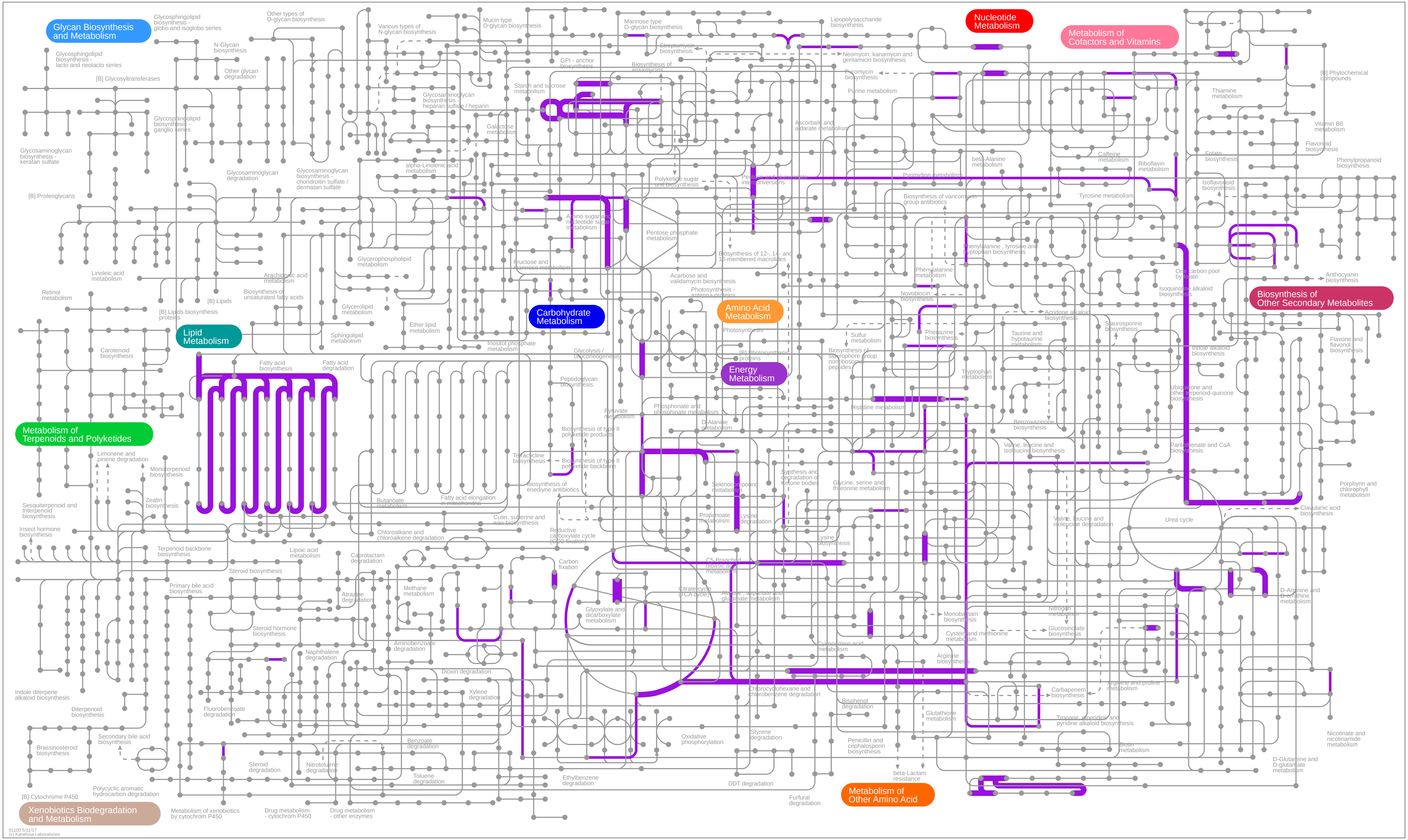

Figure S3k. Metabolic pathways overrepresented in *Firmicutes* cell envelopes of CDIC microbiota, compared to controls.

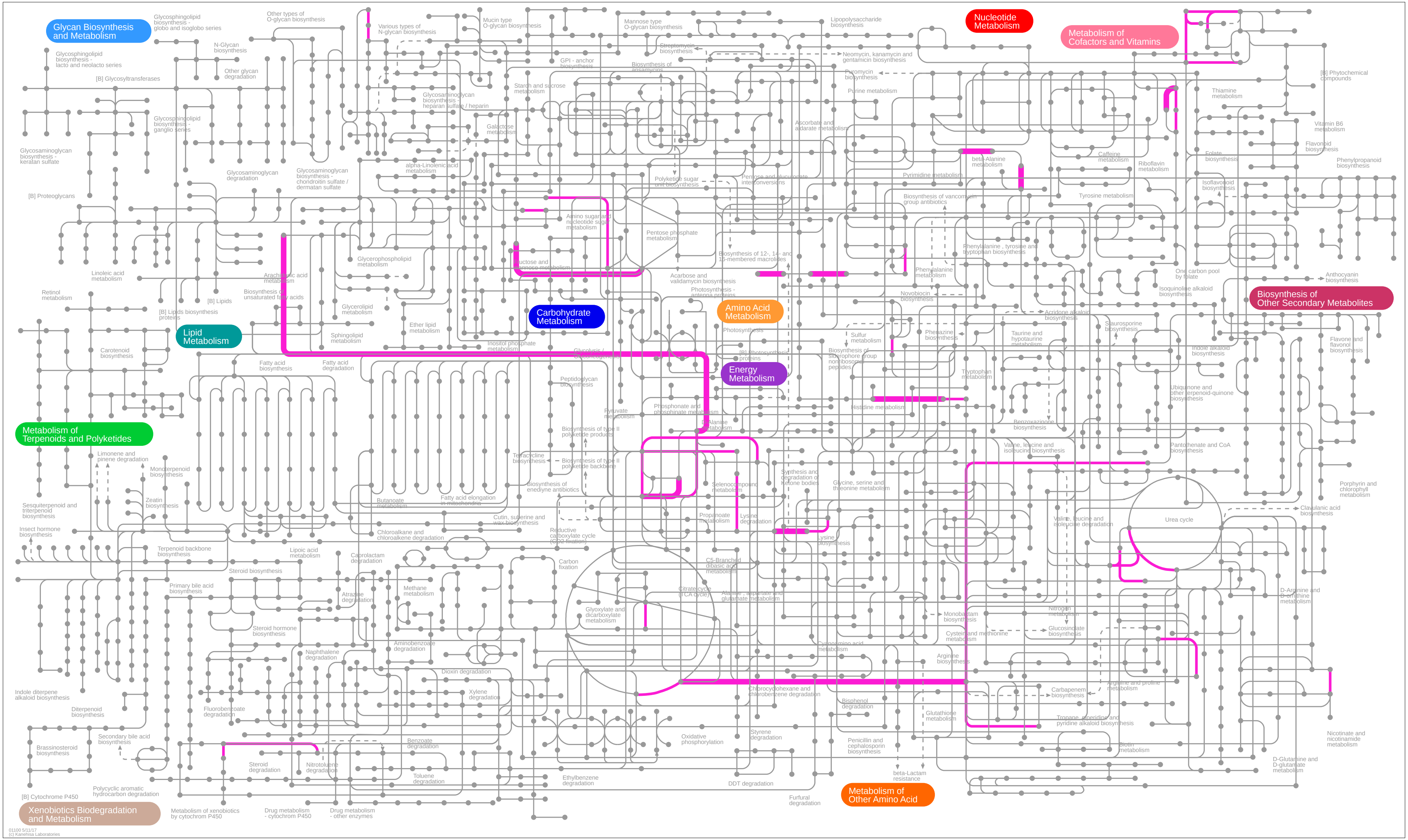

Figure S3l. Metabolic pathways overrepresented in *Firmicutes* cell envelopes of CDC microbiota, compared to controls.

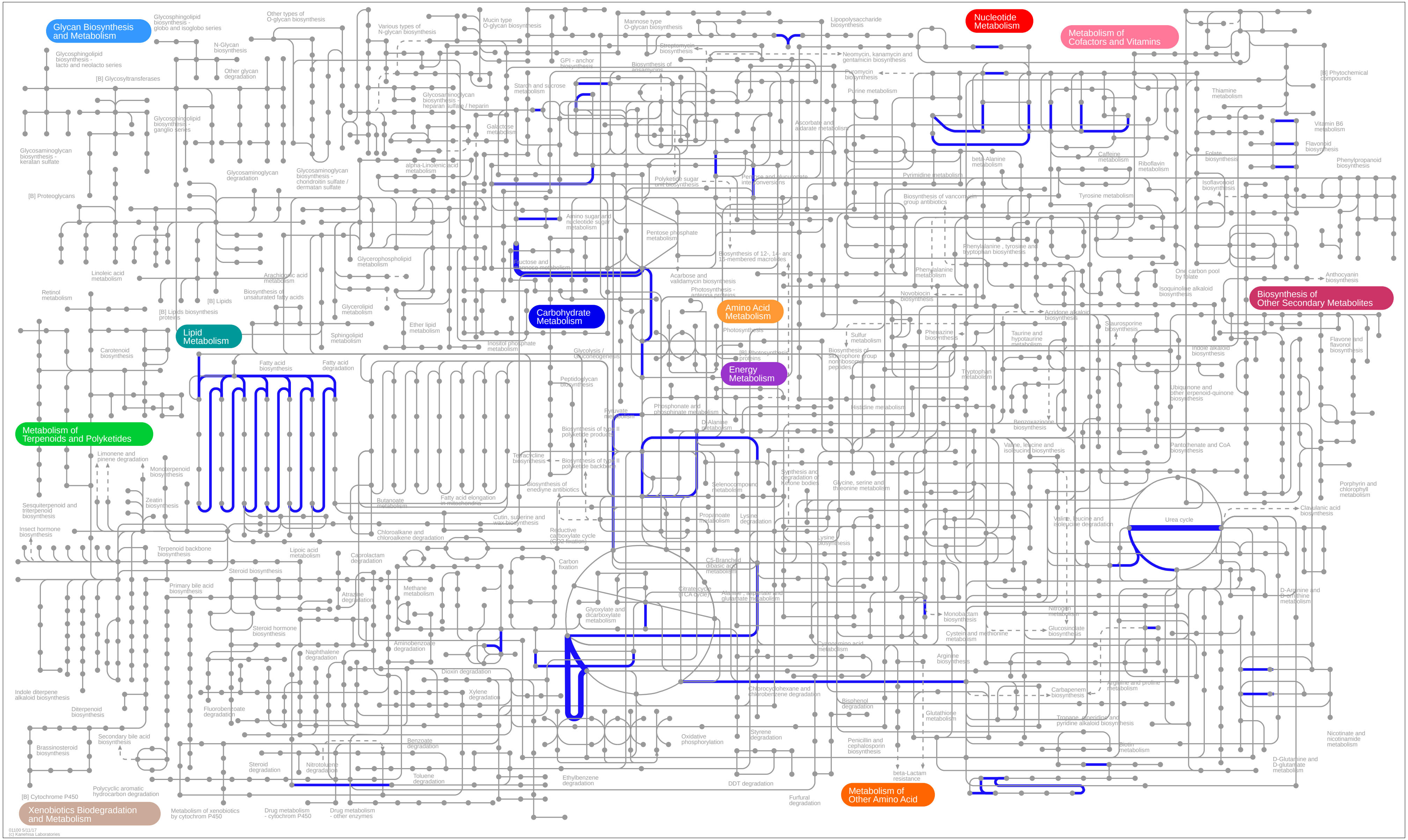

Figure S3m. Metabolic pathways overrepresented in *Firmicutes* cell envelopes of UC microbiota, compared to controls.

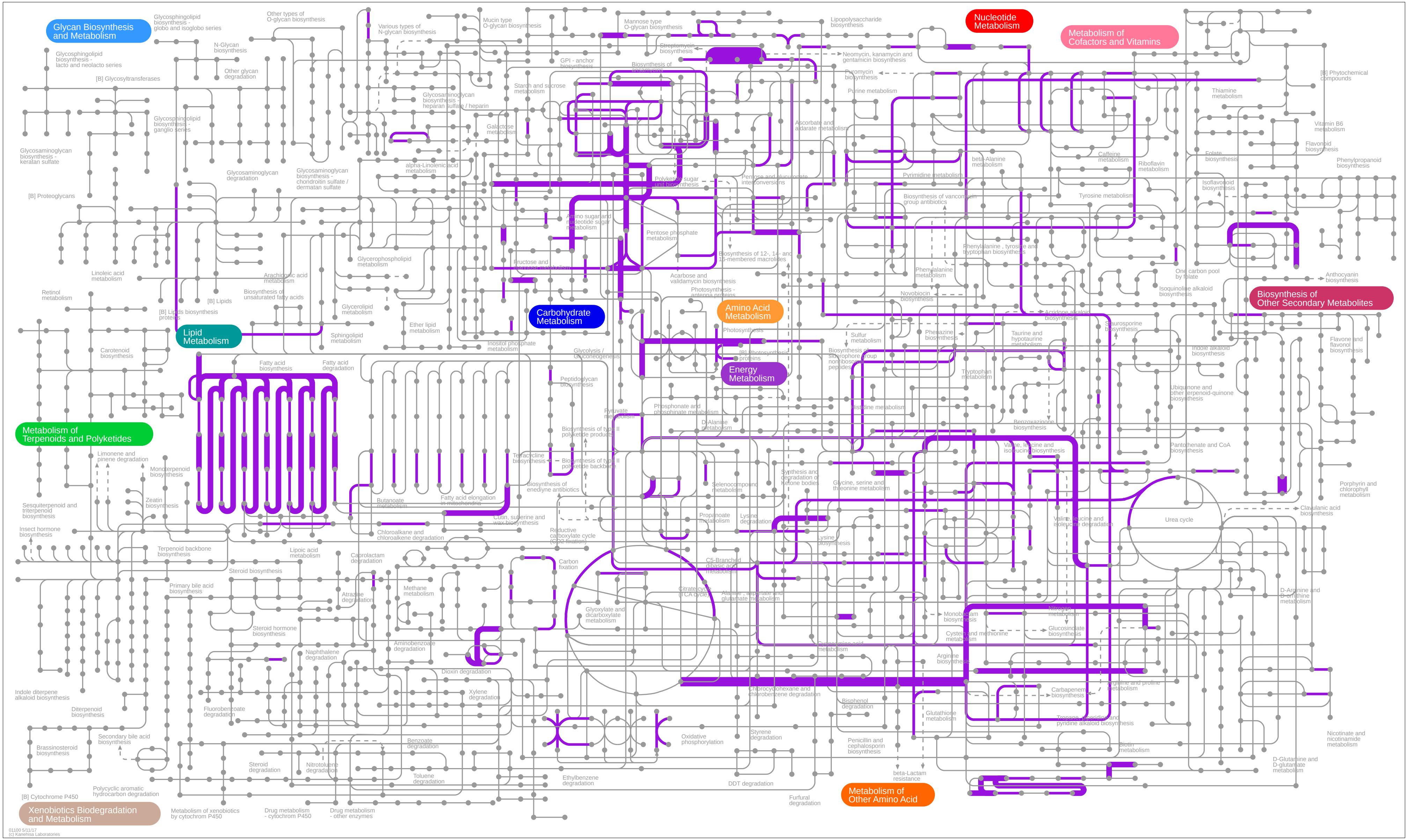

Figure S3n. Metabolic pathways underrepresented in *Bacteroidetes* cell envelopes of CDIC microbiota, compared to controls.

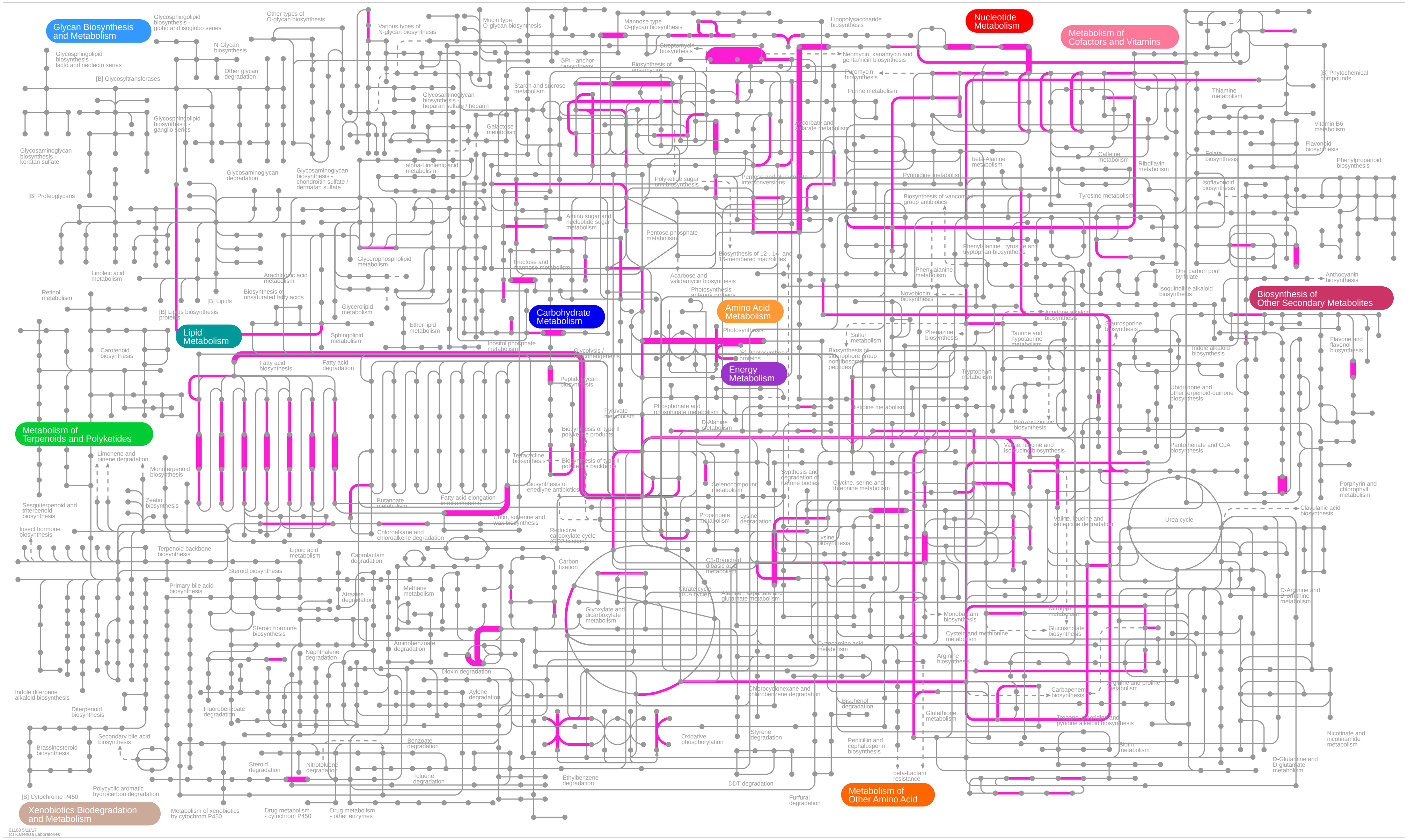

Figure S3o. Metabolic pathways underrepresented in *Bacteroidetes* cell envelopes of CDC microbiota, compared to controls.

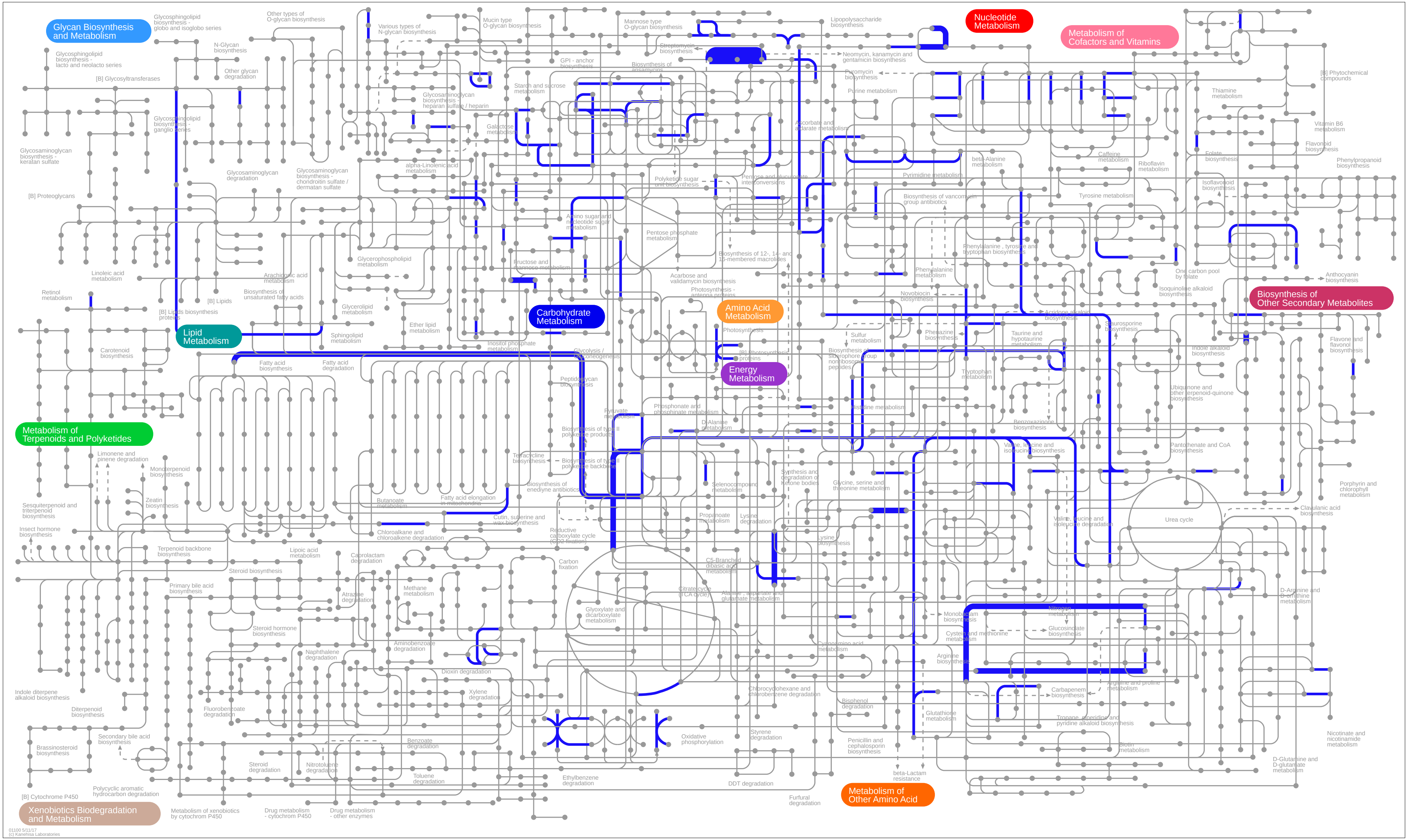

Figure S3p. Metabolic pathways underrepresented in *Bacteroidetes* cell envelopes of UC microbiota, compared to controls.

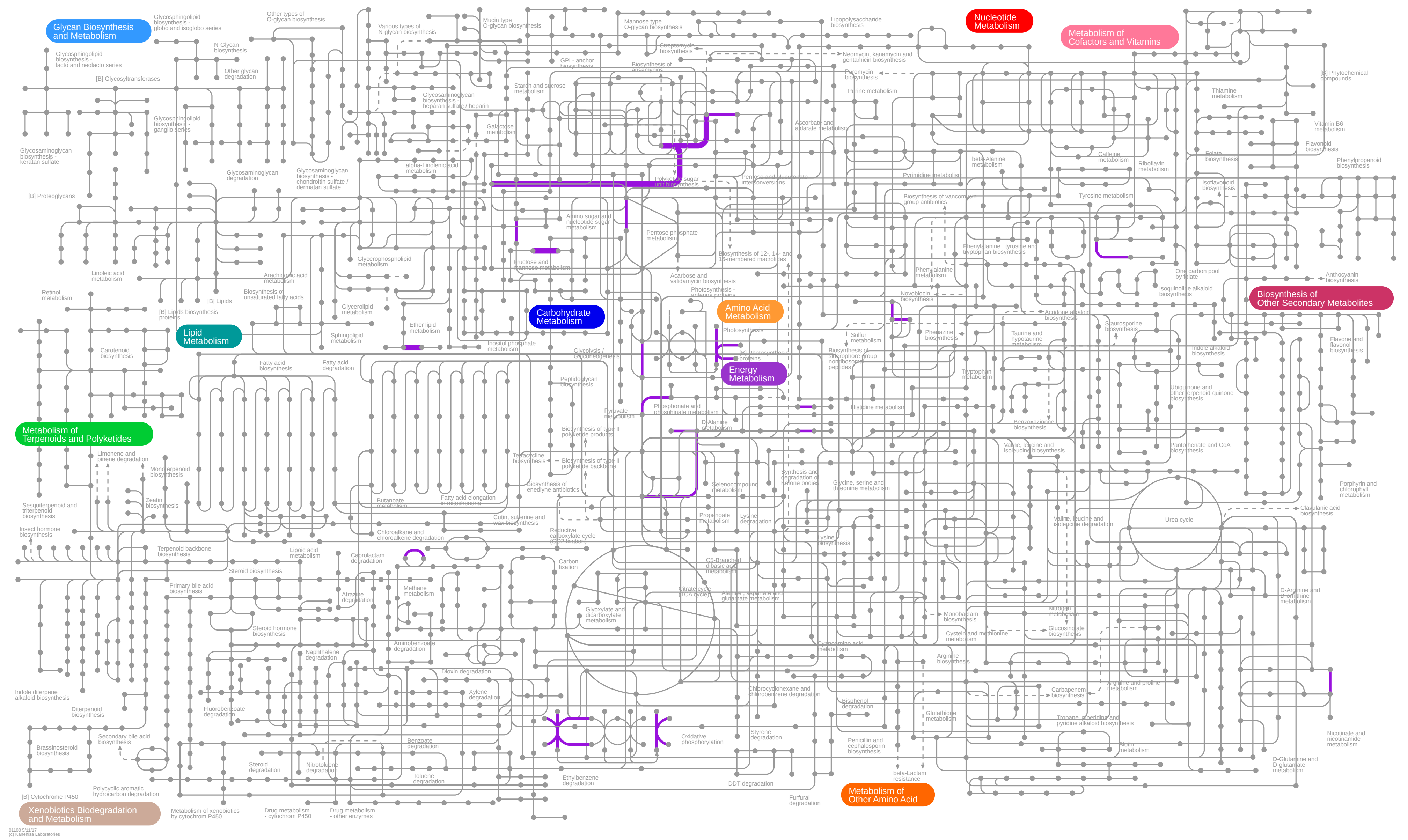

Figure S3q. Metabolic pathways overrepresented in *Bacteroidetes* cell envelopes of CDIC microbiota, compared to controls.

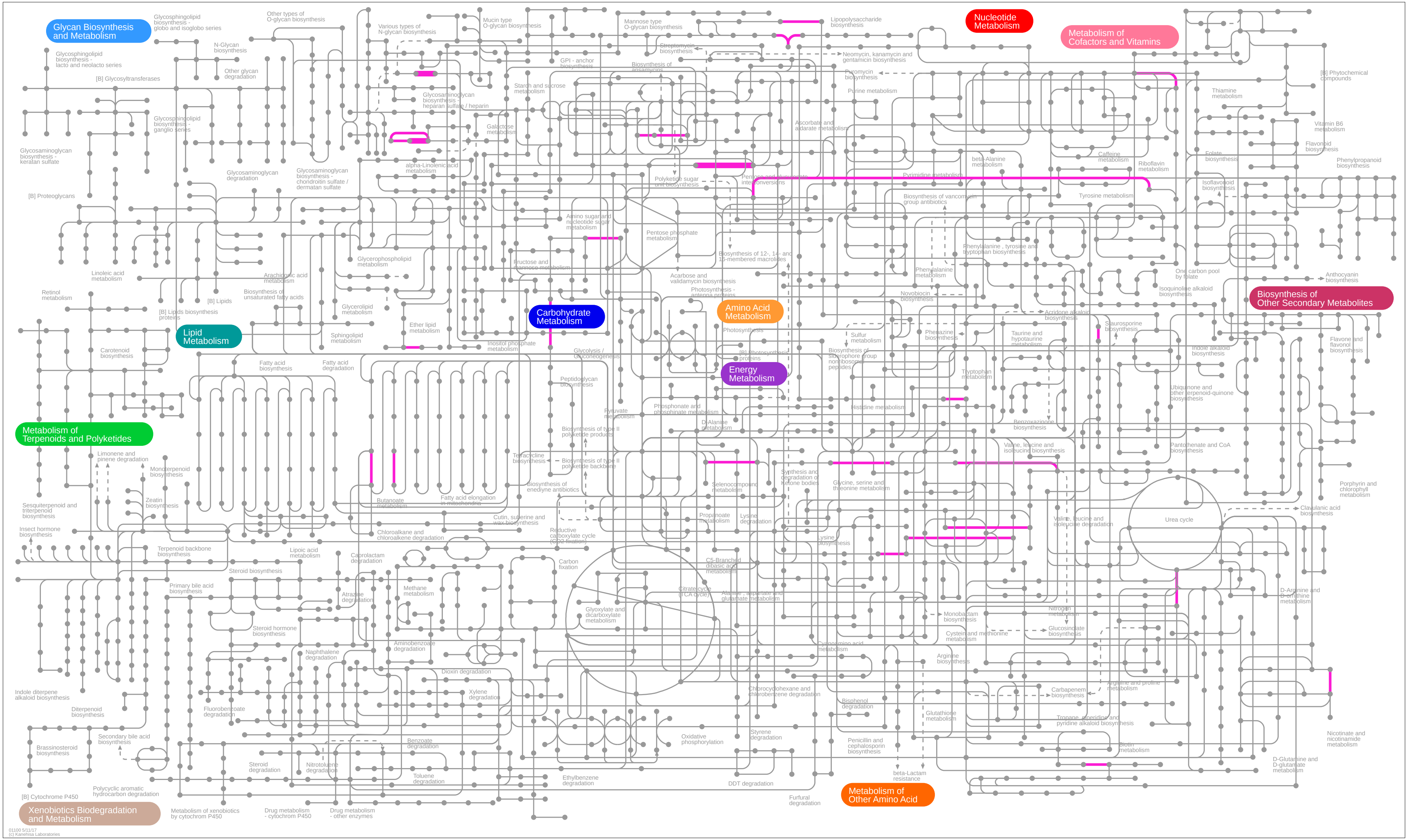

Figure S3r. Metabolic pathways overrepresented in *Bacteroidetes* cell envelopes of CDC microbiota, compared to controls.

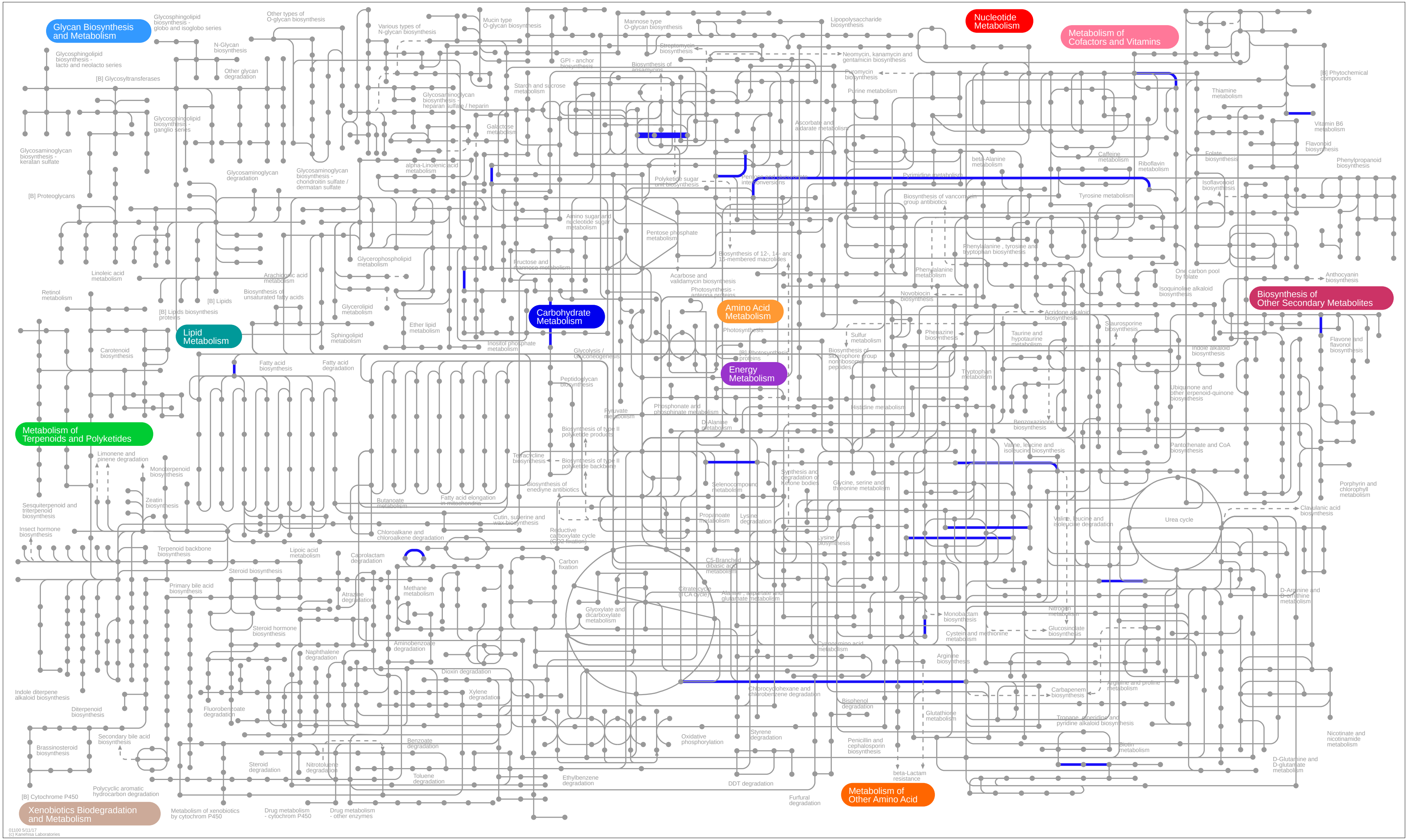

Figure S3s. Metabolic pathways overrepresented in *Bacteroidetes* cell envelopes of UC microbiota, compared to controls.

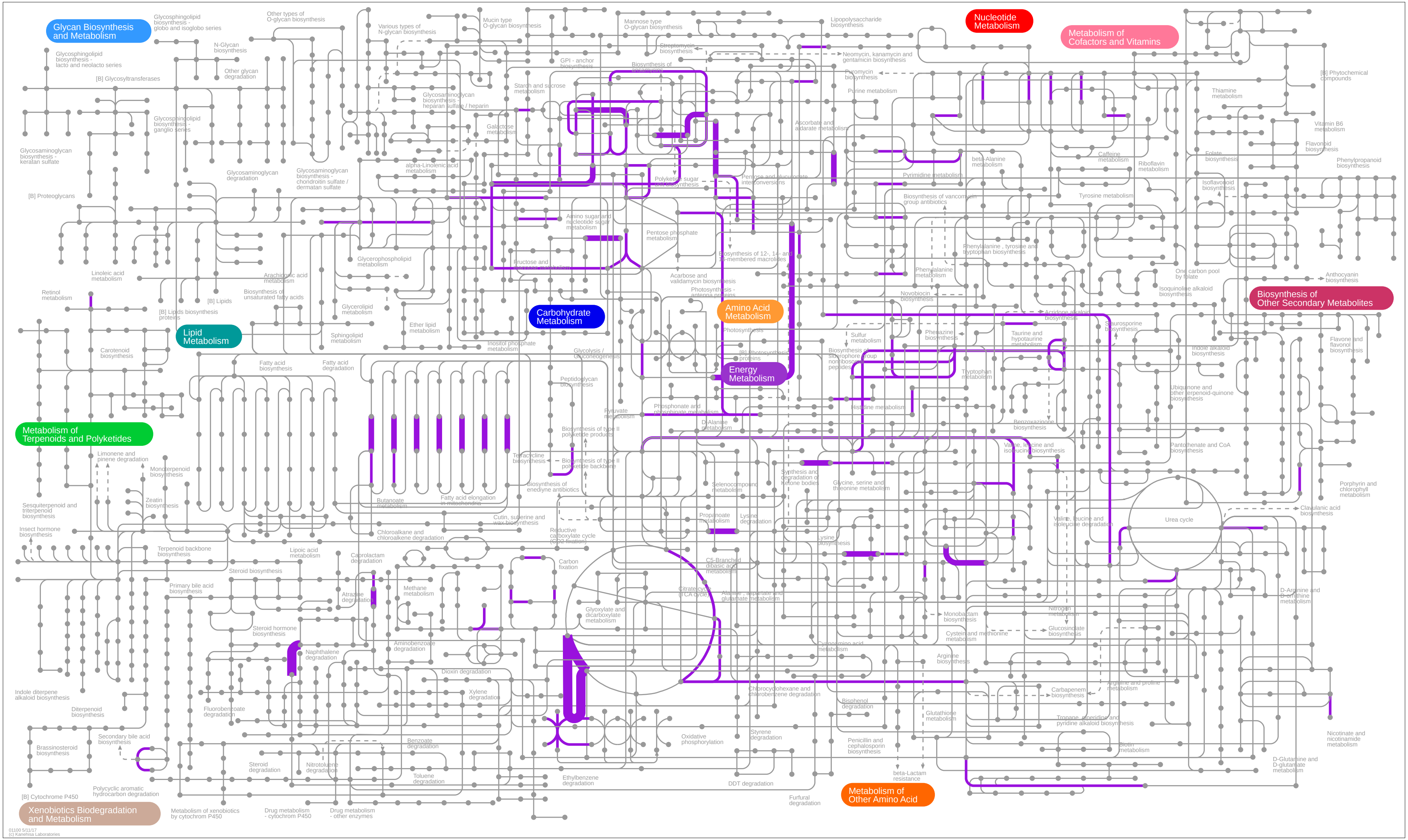

Figure S3t. Metabolic pathways underrepresented in *Actinobacteria* cell envelopes of CDIC microbiota, compared to controls.

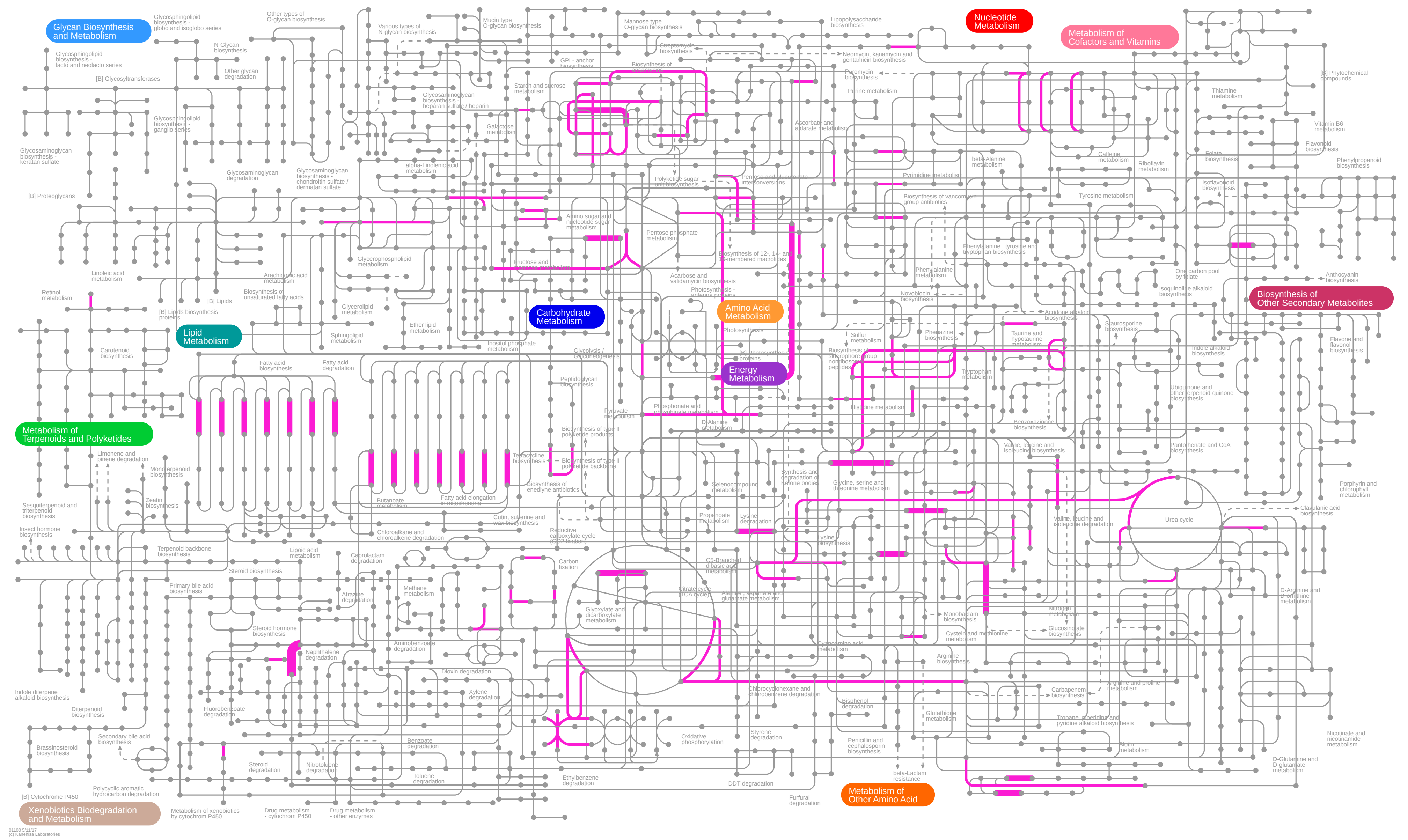

Figure S3u. Metabolic pathways underrepresented in *Actinobacteria* cell envelopes of CDC microbiota, compared to controls.

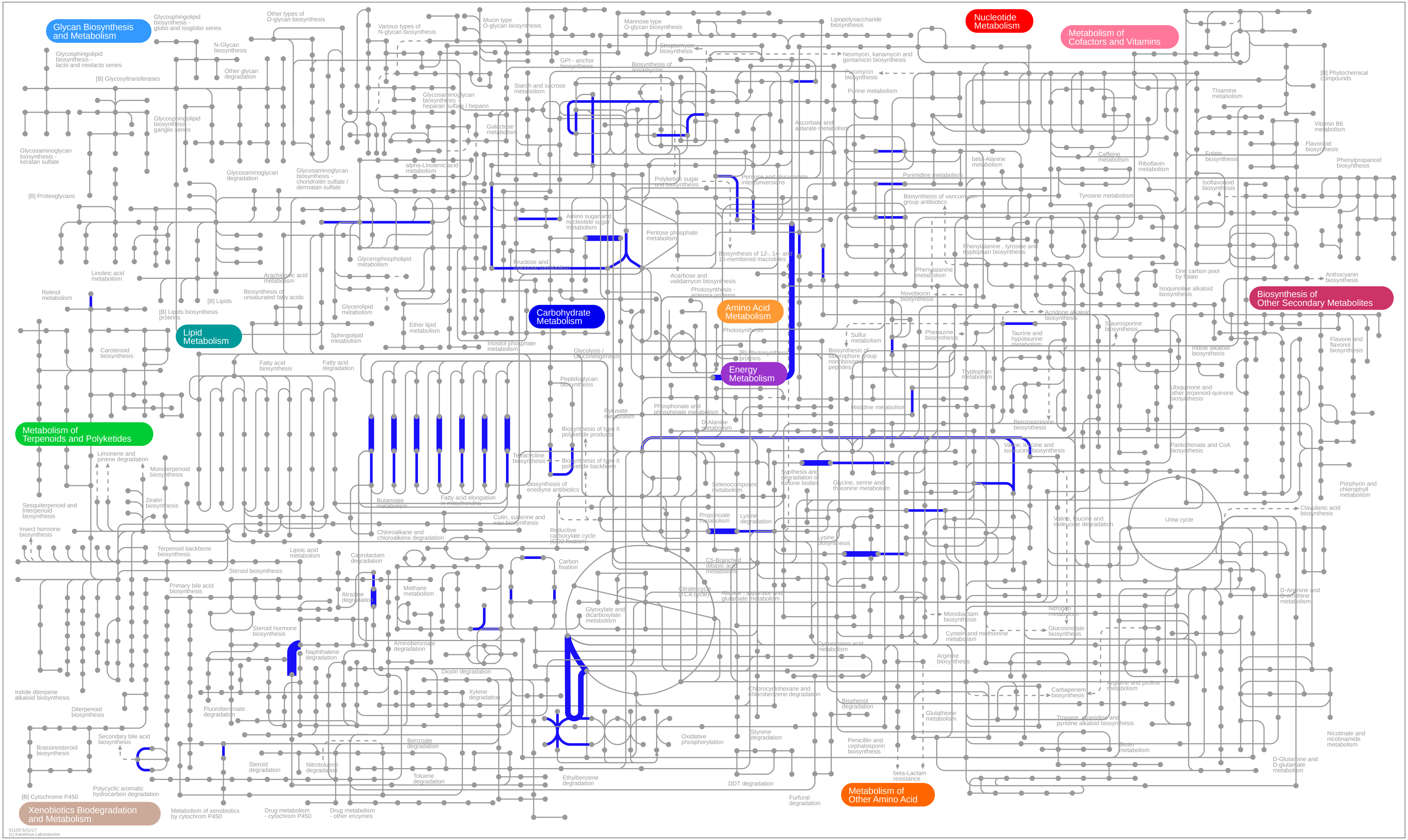

Figure S3v. Metabolic pathways underrepresented in *Actinobacteria* cell envelopes of UC microbiota, compared to controls.

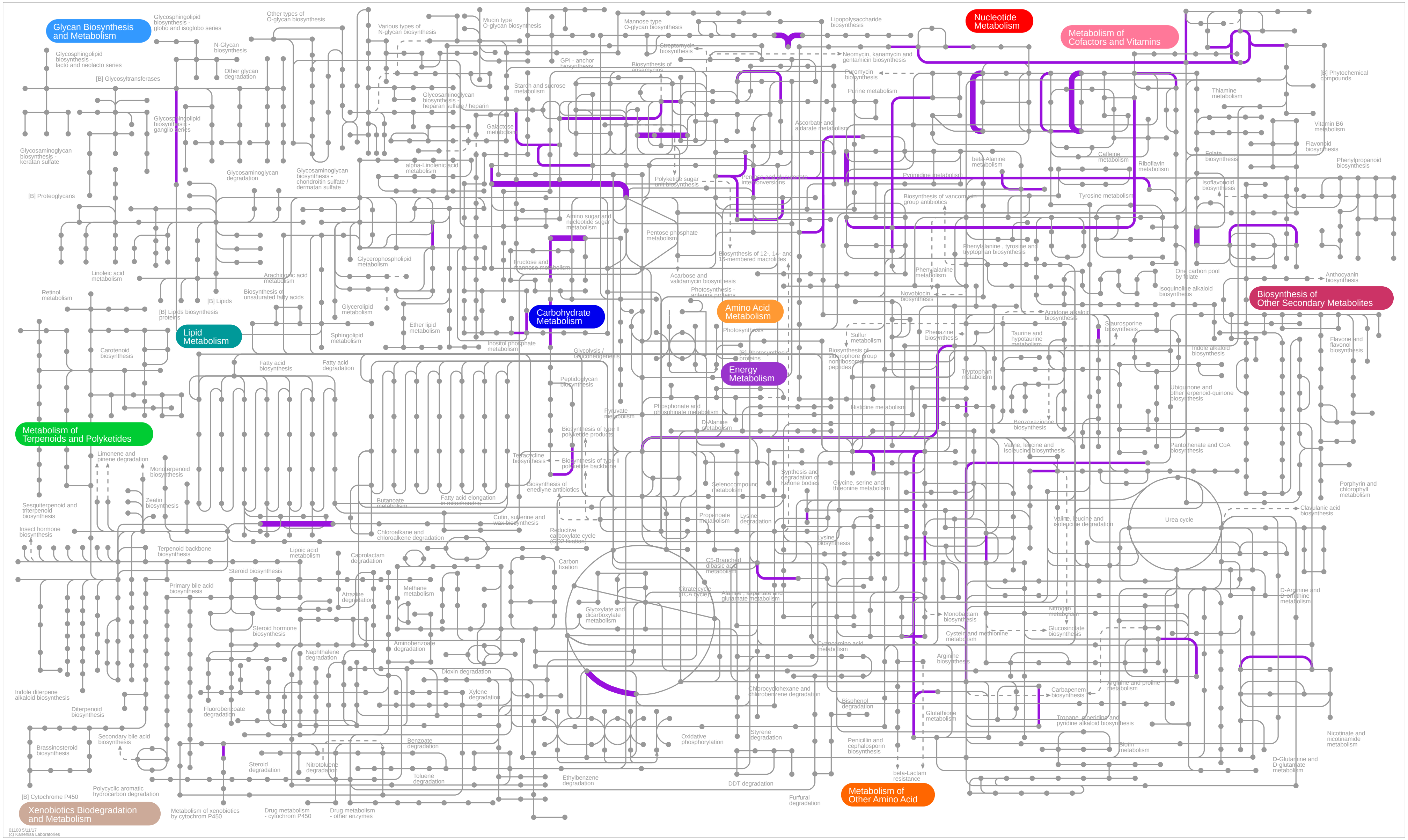

Figure S3w. Metabolic pathways overrepresented in *Actinobacteria* cell envelopes of CDIC microbiota, compared to controls.

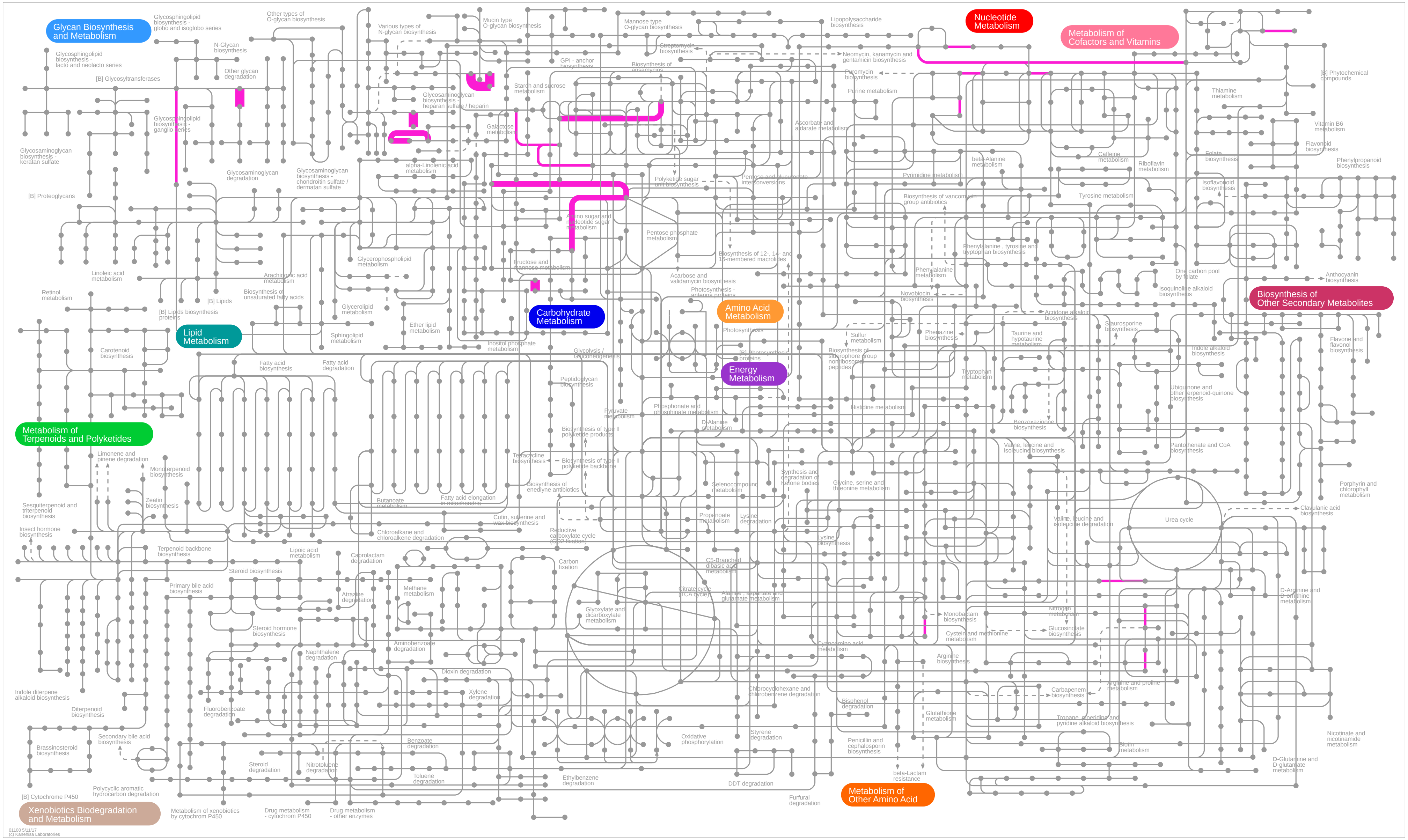

Figure S3x. Metabolic pathways overrepresented in *Actinobacteria* cell envelopes of CDC microbiota, compared to controls.

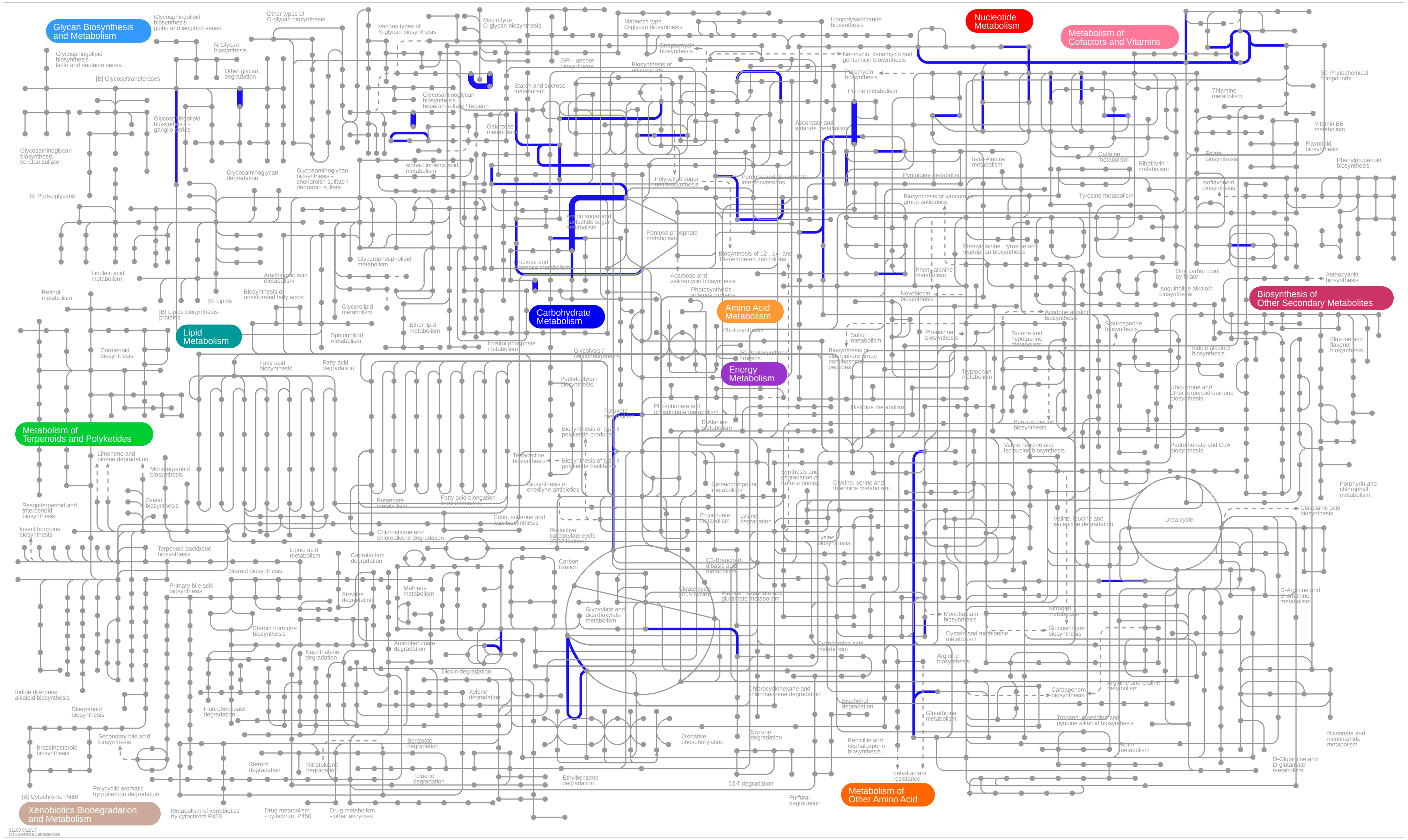

Figure S3y. Metabolic pathways overrepresented in *Actinobacteria* cell envelopes of UC microbiota, compared to controls.
