# Supplementary material for: Modern Metaproteomics: A Unique Tool to Characterize the Active Microbiome in Health and Diseases, and Pave the Road towards New Biomarkers—Example of Crohn’s Disease and Ulcerative Colitis Flare-Ups
Source: Cells. 2022 Apr 14;11(8):1340. doi: 10.3390/cells11081340 (PMC9028112; doi:10.3390/cells11081340)
Supplement: Supplementary file 1 [file cells-11-01340-s001.zip › Table_S1_statistics_report_for_Figure2.pdf]

Table S1. Wilcoxon tests with Benjamini-Hochberg stepwise adjustment for multiple pairwise comparisons between clinical groups (adjusted p-value threshold = 0.05). Variables in the left- and right-hand column are those illustrated on Figure 2a and Figure 2b, respectively. Significant pairwise comparisons are in bold characters.

subgrp.ALL

	CDC	CDIC	CTRL
CDIC	0.60952	-	-
CTRL	**0.00032**	**0.00991**	-
UC	0.28693	0.28693	**5.8e-07**

subgrp.bact

	CDC	CDIC	CTRL
CDIC	0.4762	-	-
CTRL	**0.0027**	**0.0099**	-
UC	0.1556	0.1186	**8.3e-08**

subgrp.phylum

	CDC	CDIC	CTRL
CDIC	0.47619	-	-
CTRL	**0.00032**	**0.00991**	-
UC	0.15560	0.11863	**8.3e-08**

subgrp.class

	CDC	CDIC	CTRL
CDIC	0.47619	-	-
CTRL	**0.00032**	**0.00991**	-
UC	0.15560	0.11863	**8.3e-08**

subgrp.order

	CDC	CDIC	CTRL
CDIC	0.47619	-	-
CTRL	**0.00032**	**0.00991**	-
UC	0.15560	0.11863	**8.3e-08**

subgrp.family

	CDC	CDIC	CTRL
CDIC	0.4762	-	-
CTRL	**0.0024**	**0.0187**	-
UC	0.1556	0.1186	**2.1e-05**

subgrp.genus

	CDC	CDIC	CTRL
CDIC	0.47619	-	-
CTRL	**0.00032**	**0.00991**	-
UC	0.13121	0.11863	**8.3e-08**

subgrp.species

	CDC	CDIC	CTRL
CDIC	0.60952	-	-
CTRL	**0.00056**	**0.00330**	-
UC	0.13121	0.11863	**8.3e-08**

subgrp.human

	CDC	CDIC	CTRL
CDIC	0.4762	-	-
CTRL	**0.0200**	**0.0012**	-
UC	**0.0391**	**0.0157**	**8.3e-08**

n.phyla

	CDC	CDIC	CTRL
CDIC	0.34	-	-
CTRL	0.34	0.83	-
UC	0.87	0.34	0.34

n.classes

	CDC	CDIC	CTRL
CDIC	0.293	-	-
CTRL	0.227	0.654	-
UC	0.639	0.186	0.057

n.orders

	CDC	CDIC	CTRL
CDIC	0.425	-	-
CTRL	0.199	0.849	-
UC	0.548	0.199	0.055

n.families

	CDC	CDIC	CTRL
CDIC	0.20	-	-
CTRL	0.14	0.70	-
UC	0.70	0.20	0.14

n.genera

	CDC	CDIC	CTRL
CDIC	0.4229	-	-
CTRL	0.1650	0.6029	-
UC	0.3111	0.3111	**0.0071**

n.species

	CDC	CDIC	CTRL
CDIC	0.60952	-	-
CTRL	0.06676	0.25362	-
UC	0.19450	0.15817	**0.00016**

n.ko

	CDC	CDIC	CTRL
CDIC	0.2143	-	-
CTRL	**0.0092**	0.4495	-
UC	0.2143	**0.0092**	**8e-06**
