# Supplementary material for: Modern Metaproteomics: A Unique Tool to Characterize the Active Microbiome in Health and Diseases, and Pave the Road towards New Biomarkers—Example of Crohn’s Disease and Ulcerative Colitis Flare-Ups
Source: Cells. 2022 Apr 14;11(8):1340. doi: 10.3390/cells11081340 (PMC9028112; doi:10.3390/cells11081340)
Supplement: Supplementary file 1 [file cells-11-01340-s001.zip › Table_S2_statistics_report_for_Figure3.pdf]

Table S2. Wilcoxon tests with Benjamini-Hochberg stepwise adjustment for multiple pairwise comparisons between clinical groups (adjusted p-value threshold = 0.05. Variables are those illustrated on Figure 3, i.e. sum of specific spectra (SC) per species, then packed into phyla. Only phyla for which Kruskal-Wallis test was significant are reported. Significant pairwise comparisons are in bold characters.

Abundance

sum_SC in the phylum *Firmicutes*

	CDC	CDIC	CTRL
CDIC	0.47619	-	-
CTRL	**0.00056**	**0.04396**	-
UC	**0.04396**	0.12157	**8.3e-08**

sum_SC in the phylum *Proteobacteria*

	CDC	CDIC	CTRL
CDIC	**0.0190**	-	-
CTRL	0.5265	**0.0087**	-
UC	0.7181	**0.0078**	0.2099

sum_SC in the phylum *Bacteroidetes*

	CDC	CDIC	CTRL
CDIC	0.4229	-	-
CTRL	0.3462	0.6167	-
UC	0.3057	0.2373	**0.0021**

sum_SC in unclassified bacteria

	CDC	CDIC	CTRL
CDIC	0.476	-	-
CTRL	0.166	0.166	-
UC	0.476	0.396	**0.015**

sum_SC for human proteins

	CDC	CDIC	CTRL
CDIC	0.257	-	-
CTRL	**0.010**	0.146	-
UC	0.061	**0.016**	**2.5e-06**

number_Subgroups in the phylum *Firmicutes*

	CDC	CDIC	CTRL
CDIC	0.7619	-	-
CTRL	**0.0024**	**0.0058**	-
UC	0.0933	0.1522	**1.7e-07**

number_Subgroups in the phylum *Proteobacteria*

	CDC	CDIC	CTRL
CDIC	**0.0278**	-	-
CTRL	0.3416	**0.0087**	-
UC	0.7104	**0.0087**	0.1056

number_Subgroups in the phylum *Bacteroidetes*

	CDC	CDIC	CTRL
CDIC	0.42	-	-
CTRL	0.25	0.75	-
UC	0.42	0.25	**0.03**

number_Subgroups in unclassified bacteria

	CDC	CDIC	CTRL
CDIC	0.6689	-	-
CTRL	0.0643	0.1033	-
UC	0.4066	0.4066	**0.0046**

number_Subgroups human proteins

	CDC	CDIC	CTRL
CDIC	0.4762	-	-
CTRL	**0.0224**	**0.0012**	-
UC	**0.0391**	**0.0157**	**8.3e-08**

Diversity
