# Supplementary figures and images for: Modern Metaproteomics: A Unique Tool to Characterize the Active Microbiome in Health and Diseases, and Pave the Road towards New Biomarkers—Example of Crohn’s Disease and Ulcerative Colitis Flare-Ups
Source: Cells. 2022 Apr 14;11(8):1340. doi: 10.3390/cells11081340 (PMC9028112; doi:10.3390/cells11081340)

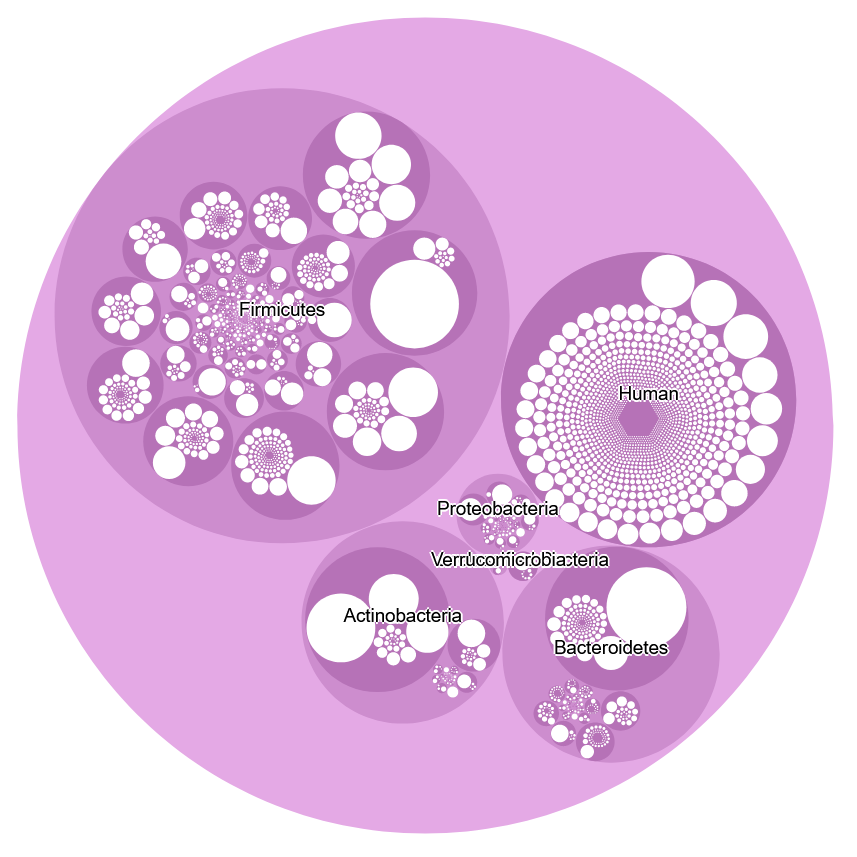

Supplement: Supplementary file 1 [file cells-11-01340-s001.zip › Figure_S2_circlePacke/Circlepacke_CDC_Fig3.png]

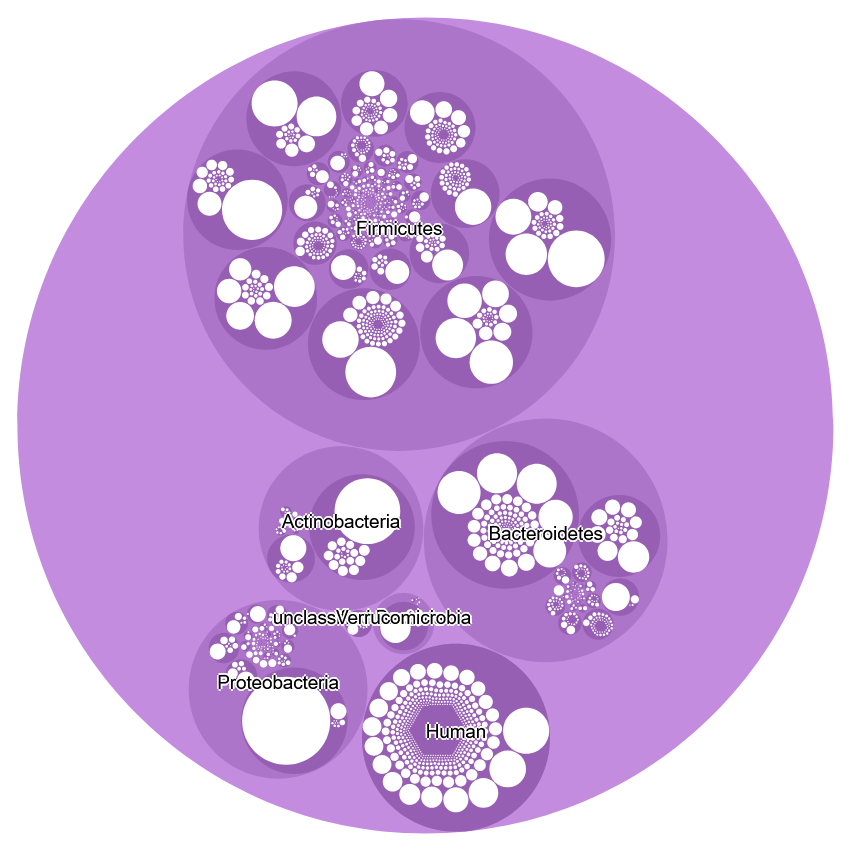

Supplement: Supplementary file 1 [file cells-11-01340-s001.zip › Figure_S2_circlePacke/Circlepacke_CDIC_Fig3.png]

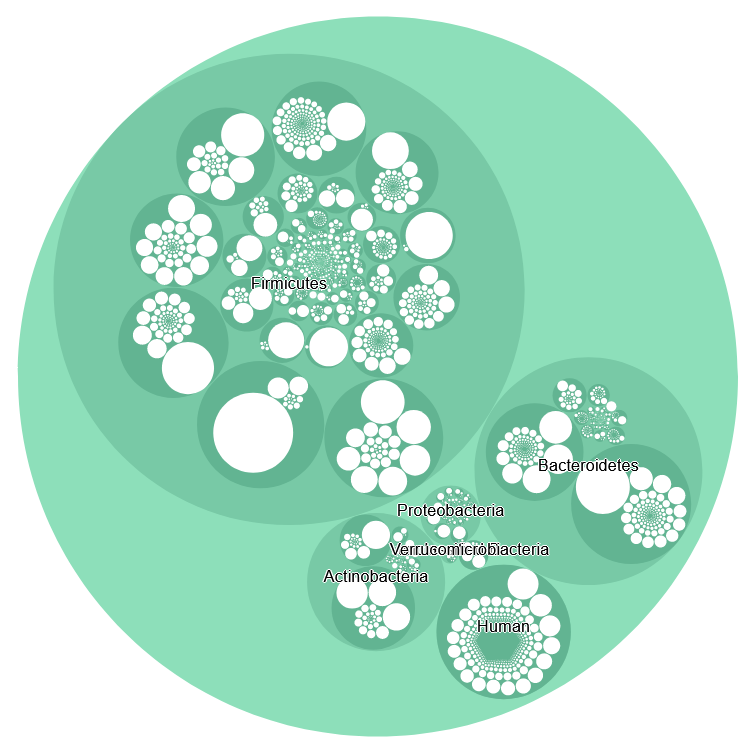

Supplement: Supplementary file 1 [file cells-11-01340-s001.zip › Figure_S2_circlePacke/Circlepacke_CTRL_Fig3.png]

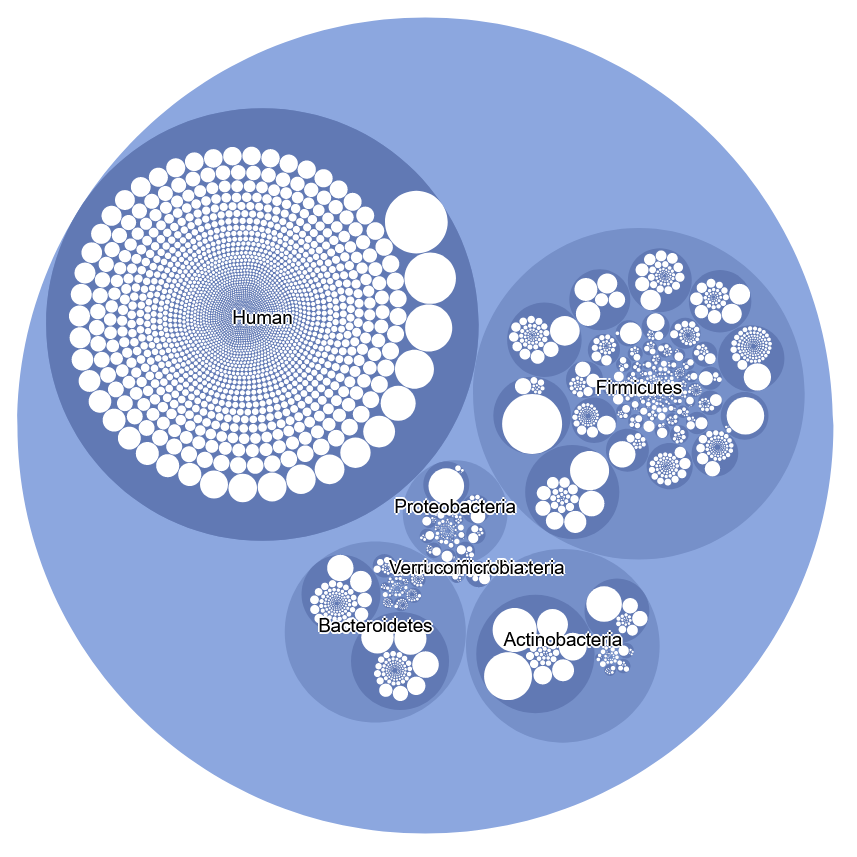

Supplement: Supplementary file 1 [file cells-11-01340-s001.zip › Figure_S2_circlePacke/Circlepacke_UC_Fig3.png]
